# Cervical Cancer Screening Cascade for women living with HIV: A cohort study from Zimbabwe

**DOI:** 10.1371/journal.pgph.0000156

**Published:** 2022-02-02

**Authors:** Katayoun Taghavi, Ardele Mandiriri, Tinei Shamu, Eliane Rohner, Lukas Bütikofer, Serra Asangbeh, Tsitsi Magure, Cleophas Chimbetete, Matthias Egger, Margaret Pascoe, Julia Bohlius

**Affiliations:** 1 Institute of Social and Preventive Medicine, University of Bern, Bern, Switzerland; 2 The Graduate School for Cellular and Biomedical Sciences of the University of Bern, Bern, Switzerland; 3 Newlands Clinic, Harare, Zimbabwe; 4 The Graduate School for Health Sciences of the University of Bern, Bern, Switzerland; 5 CTU, University of Bern, Bern, Switzerland; 6 Swiss Tropical and Public Health Institute, Basel, Switzerland; 7 Centre for Infectious Disease Epidemiology and Research, School of Public Health, University of Cape Town, Cape Town, South Africa; 8 Population Health Sciences, Bristol Medical School, University of Bristol, Bristol, United Kingdom; 9 University of Basel, Basel, Switzerland; University of Alabama at Birmingham, UNITED STATES

## Abstract

Countries with high HIV prevalence, predominantly in sub-Sahahran Africa, have the highest cervical cancer rates globally. HIV care cascades successfully facilitated the scale-up of antiretroviral therapy. A cascade approach could similarly succeed to scale-up cervical cancer screening, supporting WHO’s goal to eliminate cervical cancer. We defined a Cervical Cancer Screening Cascade for women living with HIV (WLHIV), evaluating the continuum of cervical cancer screening integrated into an HIV clinic in Zimbabwe. We included WLHIV aged ≥18 years enrolled at Newlands Clinic in Harare from June 2012–2017 and followed them until June 2018. We used a cascade approach to evaluate the full continuum of secondary prevention from screening to treatment of pre-cancer and follow-up. We report percentages, median time to reach cascade stages, and cumulative incidence at two years with 95% confidence intervals (CI). We used univariable Cox proportional hazard regressions to calculate cause-specific hazard ratios with 95% CIs for factors associated with completing the cascade stages. We included 1624 WLHIV in the study. The cumulative incidence of cervical screening was 85.4% (95% CI 83.5–87.1) at two years. Among the 396 WLHIV who received screen-positive tests in the study, the cumulative incidence of treatment after a positive screening test was 79.5% (95% CI 75.1–83.2) at two years. The cumulative incidence of testing negative at re-screening after treatment was 36.1% (95% CI 31.2–40.7) at two years. Using a cascade approach to evaluate the full continuum of cervical cancer screening, we found less-than 80% of WLHIV received treatment after screen-positive tests and less-than 40% were screen-negative at follow-up. Interventions to improve linkage to treatment for screen-positive WLHIV and studies to understand the clinical significance of screen-positive tests at follow-up among WLHIV are needed. These gaps in the continuum of care must be addressed in order to prevent cervical cancer.

## Introduction

Countries with high HIV prevalence, predominantly in sub-Saharan Africa, have the highest cervical cancer rates in the world [1, 2]. Many sub-Saharan African countries introduced pilot screening programs in the 1990s, yet, cervical cancer remains the most common cancer among women [3, 4]. In Zimbabwe, over 2000 cervical cancer cases are recorded each year, and this is considered a significant under-estimation [5–7]. Furthermore, women with cervical cancer usually present at late stages of the disease, resulting in high morbidity and mortality [8]. Women living with HIV (WLHIV) are at higher risk of cancer and persistant or recurrent precancerous lesions than the general population, so follow-up and repeated screenings are especially important. For this high-risk group, the integration of cervical cancer screening and management into HIV care may be beneficial [1].

Cervical cancer incidence and mortality decrease when screening programs engage and retain women from screening to treatment to follow-up along a care continuum [9, 10]. The HIV care cascade has been successfully used to scale-up of antiretroviral therapy (ART) [11–15]. We believe the cascade approach could be similarly used to scale-up cervical cancer screening, supporing the WHO goal to eliminate cervical cancer. It would especially benefit WLHIV who are at higher risk of persistant human papillomavirus (HPV) infection and require repeat treatments [16–19].

In this study, we applied the cascade approach, to cervical cancer screening for WLHIV. We defined and assessed a Cervical Cancer Screening Cascade for WLHIV enrolled at an ART clinic with integrated screening services in Harare, Zimbabwe and explored patient factors associated with retention through stages of the cascade.

## Methods

### Study design

This retrospective cohort study used routinely collected data from an HIV care and treatment clinic with integrated cervical screening in Harare, Zimbabwe.

### Study site

The Newlands Clinic (https://www.newlandsclinic.org.zw) is an HIV/AIDS referral clinic run by a Swiss charitable organization (S1 Text). The integrated cervical screening program was established in 2011, using primarily visual inspection with acetic acid and cervicography (VIAC) (S1 Text). A Papanicolaou (PAP) test is performed when the transformation zone is not visible, and biopsies are taken of ambiguous lesions or those suspicious of cancer. Nurses treat small precancerous lesions with cryotherapy on the same day as screening (S1 Text). Once a week, a gynecologist performs loop electrosurgical excision procedures (LEEP) and provides a second opinion on cervical images if needed. Newlands Clinic adheres to the following screening schedule: first screening test within 12 months of enrollment and re-screen within 12 months if the first test is negative. For a positive test, women are treated within six months and re-screened within six months of treatment.

### Eligibility criteria

We included all women aged 18 years or older who had their first ART clinic visit between June 2012 and June 2017 (“women in care”). We allowed for a one-year follow-up, with data collection ending in June 2018. The cohort was identified through the clinic’s electronic patient-care system.

### Cascade stages

**Fig 1:**
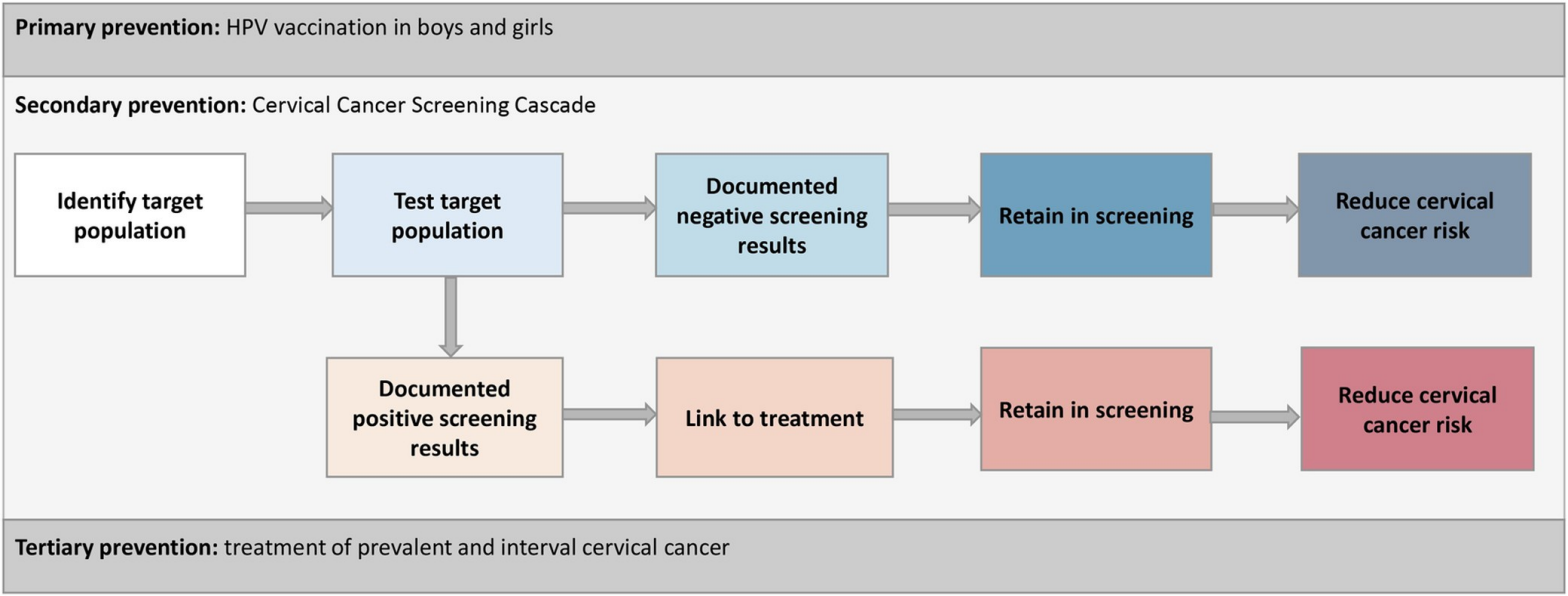
Conceptual model for a Cervical Cancer Screening Cascade nested in the three pillars of cervical cancer prevention: Primary, secondary and tertiary. The conceptual model considers the three pillars of cervical cancer prevention: primary, secondary and tertiary. A cascade for secondary cervical cancer prevention is detailed and is the focus of this study. The screening arm is represented by the first line and box colors white to dark blue. The preventative treatment arm is represented by the second line and box colors orange to red. The definitions for stages of the cascade can be found in S1 Table. HPV: human papilloma virus.

We focused on secondary prevention and defined a Cervical Cancer Screening Cascade, composed of “screening” and “preventative treatment” arms (Fig 1). The screening arm follows women in HIV care through screening and re-screening after a negative screening result. Its stages are defined as the number of women: (i) in care and eligible for screening; (ii) screened for cervical cancer; (iv) with documented screen-negative results at their first screening; (v) screen-negative who re-screened; (vi) with documented screen-negative results at re-screening. The preventative treatment arm follows women in HIV care through screening and re-screening after a positive screening result. Its stages are defined as the number of women: (i) with documented screen-positive results at first screening; (ii) receiving treatment; (iii) treated who were re-screened; (iv) treated with documented screen-negative results at re-screening (Fig 1). Women may be diagnosed with cervical cancer throughout the cascade (Fig 1, defined in S1 Table). After cancer diagnosis, women are referred for tertiary prevention and exit the Cervical Cancer Screening Cascade.

### Data extraction and management

We extracted data on screening (VIAC results, PAP tests), treatment, and follow-up for all WLHIV in care (S2 Table). Data on VIAC results, HIV status including CD4 and HIV RNA viral load, demographic characteristics were exported directly from the electronic patient-care system. We manually extracted data on sexual and gynecological history, cytology and histology results, referral and re-screening history, and cervical cancer management from electronic free-text fields into an EpiData database (v4.2.0.0 EpiData—Comprehensive Data Management and Basic Statistical Analysis System. Odense Denmark). Test results for VIAC were classified as positive or negative. Indeterminate cases and those suspicious for cancer were recorded as positive. The clinic gynecologist subsequently reviewed these women. Biopsies were obtained if required, and women with cervical cancer were referred for tertiary treatment.

In our analysis, we considered positive VIAC tests or PAP tests as screen-positive, in accordance with local guidelines. We classified PAP tests as negative when they were considered clinically low-risk by Newlands guidelines, indicating repeat screening in one year. This includes cytological low-grade squamous intraepithelial lesions (LSIL) or atypical squamous cells of undetermined significance (ASC-US). We considered (i) high-grade squamous intraepithelial lesion (HSIL); (ii) atypical squamous cells-HSIL (ASC-H) cannot be excluded; (iii) LSIL, cannot exclude HSIL (LSIL-H); (iv) persistent LSIL/ASC-US; or (v) atypical glandular cells (AGC) as positive PAP tests, as these were considered clinically high-risk and would be an indication for gynecological review or treatment.

### Statistical analysis

We used descriptive statistics to report baseline patient characteristics. We report percentages for all stages of the cascade 1) with a fixed-denominator of all women enrolled in care, which remained constant throughout all stages (denominator-denominator) and 2) with a changing-denominator, where all women reaching a given stage are the denominator for the next stage (numerator-denominator). When a fixed-denominator is used, there is internal consistency, and the continuum of care across the cascade relates to the single most important denominator, women in care [14]. In contrast, when a changing-denominator is used [1, 9, 13], problems with retention (retention gaps) at different stages of the cascade are highlighted [20, 21]. We used guideline-indicated time-frames in these analyses (S1 Table).

We also calculated cumulative incidence for reaching each cascade stage. The first clinic visit was the start date of the screening arm of the cascade, and the date of a positive test at the first screening was the start of the preventative treatment arm. Observation time ended on the date a cascade arm was completed, the date a woman was determined lost to follow-up (120 days after she missed her last ART clinic appointment), the date of a woman’s cervical cancer diagnosis or death, or the end of the study period, whichever came first. We used R-package “longitudinalcascade” [22] to calculate cumulative incidences, treating different arms, death, and cervical cancer as competing risks. We report cumulative incidences as percentages with 95% confidence intervals (CI) calculated on the log-survival scale. We also report the median time to reach specific cascade steps.

We used univariable Cox proportional hazard regressions to calculate cause-specific hazard ratios with 95% CIs for factors associated with achieving cascade stages. In multivariable analyses, we included variables with p-value < 0.05 in univariable analyses and known determinants of cervical neoplasia and retention in care (i.e., parity, employment, HIV clinical stage) [23–25]. Missing data were excluded from the analysis. We used STATA v14.0 (College Station, TX: StataCorp LP) and R (R Foundation for Statistical Computing, Vienna, Austria) for all analyses and figures.

### Ethics

We obtained ethical approval from local IRBs (Medical Research Council of Zimbabwe: MRCZ/A/1336, Cantonal Ethics Committee of Bern, Switzerland: PB_2016–00273) who granted waivers of informed consent as the analyses use de-identified data collected as part of routine patient care.

## Results

### Characteristics of included women

**Fig 2:**
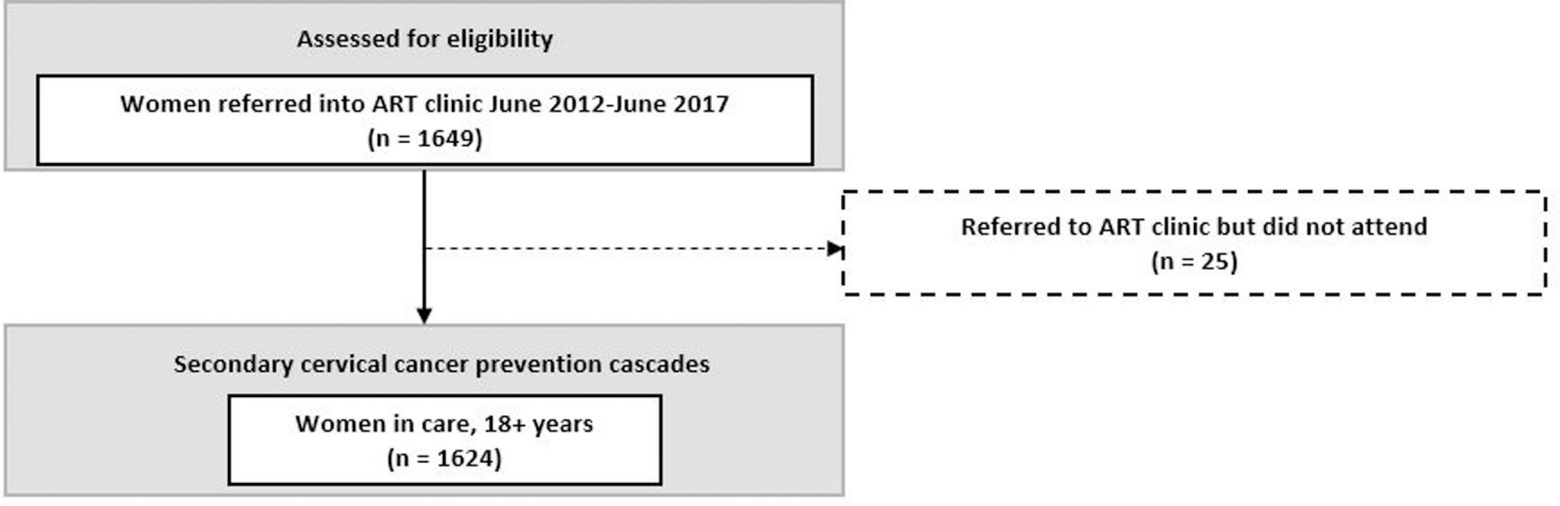
Flow diagram of women included in the analysis. ART; antiretroviral therapy, n; number of women.

Of 1649 WLHIV referred to Newlands Clinic between June 2012 and June 2017, 1624 (98.4%) attended the ART clinic and were included in the analysis (Fig 2). Their median age was 37 years (IQR 29–44) (Table 1). Most WLHIV had two children (IQR 1–4), almost half did not use contraceptives (42.4%, n = 684), and most were single, widowed or divorced (57.6%, n = 931). Most WLHIV had at least high-school education (87.3%, n = 1403) and were unemployed (54.2%, n = 875). Many were already on ART at their first clinic visit (56.4%, n = 911) and were in WHO HIV/AIDS stage 1 (55.2% n = 892).

**Table 1:** Patient characteristics at baseline.

Characteristic	n (%)^a^
**Total**	1624 (100)
**Age at entry into ART clinic: median (IQR), years**	37 (29–44)
18–21	79 (4.9)
21–25	138 (8.5)
25–45	1037 (63.9)
>45	370 (22.78)
**Sexually active at entry into ART clinic**	996 (61.3)
Not sexually active at entry into ART clinic	215 (13.2)
Missing	413 (25.4)
**Pregnant at entry into ART clinic**	86 (5.3)
Not pregnant at entry into ART clinic	1309 (81.1)
Missing	229 (14.2)
**Gravidity at entry into ART clinic: median (IQR)**	3 (2–5)
**Parity at entry into ART clinic: median (IQR)**	2 (1–4)
**Contraception at entry into ART clinic**	639 (39.6)
Not using contraception at entry into ART	684 (42.4)
Missing	301 (18.6)
**Marital status at entry into ART clinic**	
Married	686 (42.5)
Partnership	5 (0.3)
Divorced	102 (6.3)
Widowed	328 (20.3)
Single	501 (31.0)
Missing	2 (0.1)
**Education obtained at entry into ART clinic** ^**b**^	1554 (96.2)
No education obtained at entry into ART clinic	70 (4.3)
**Employment at entry into ART clinic**	558 (34.6)
Student	79 (4.9)
Unemployed	875 (54.2)
Missing	112 (6.9)
**WHO HIV/AIDS Stage at entry into ART clinic**	
1	892 (55.2)
2	260 (16.1)
3	237 (14.7)
4	148 (9.2)
Missing	87 (5.4)
**On ART at entry into ART clinic**	911 (56.4)
Not on ART at entry into ART clinic	713 (44.1)
**Nadir CD4 cell count: median (IQR), cells/mm** ^**3**^	258 (120–409)
**HIV RNA viral load at entry into ART clinic: median (IQR), copies/mm** ^**3**^	899 (28–47,969)
Missing	58 (3.6)

IQR: inter-quartile range; AIDS: acquired immunodeficiency syndrome; ART: Antiretroviral Therapy; CD4 cell: Cluster of differentiation four cells; cells/mm^3^; copies/mm^3^: copies in a cubic millimetre; HIV: human immunodeficiency virus; n: number of women living with HIV; RNA: Ribonucleic acid; WHO: World Health Organization; %: percentage; ^a^: data represent n (%) of participants unless otherwise specified, when missing data is not reported for a patient characteristic it means there was no missing data for this variable; b: education level obtained at entry into ART includes primary, high school, university and college.

### Proportions along the cascade

**Fig 3:**
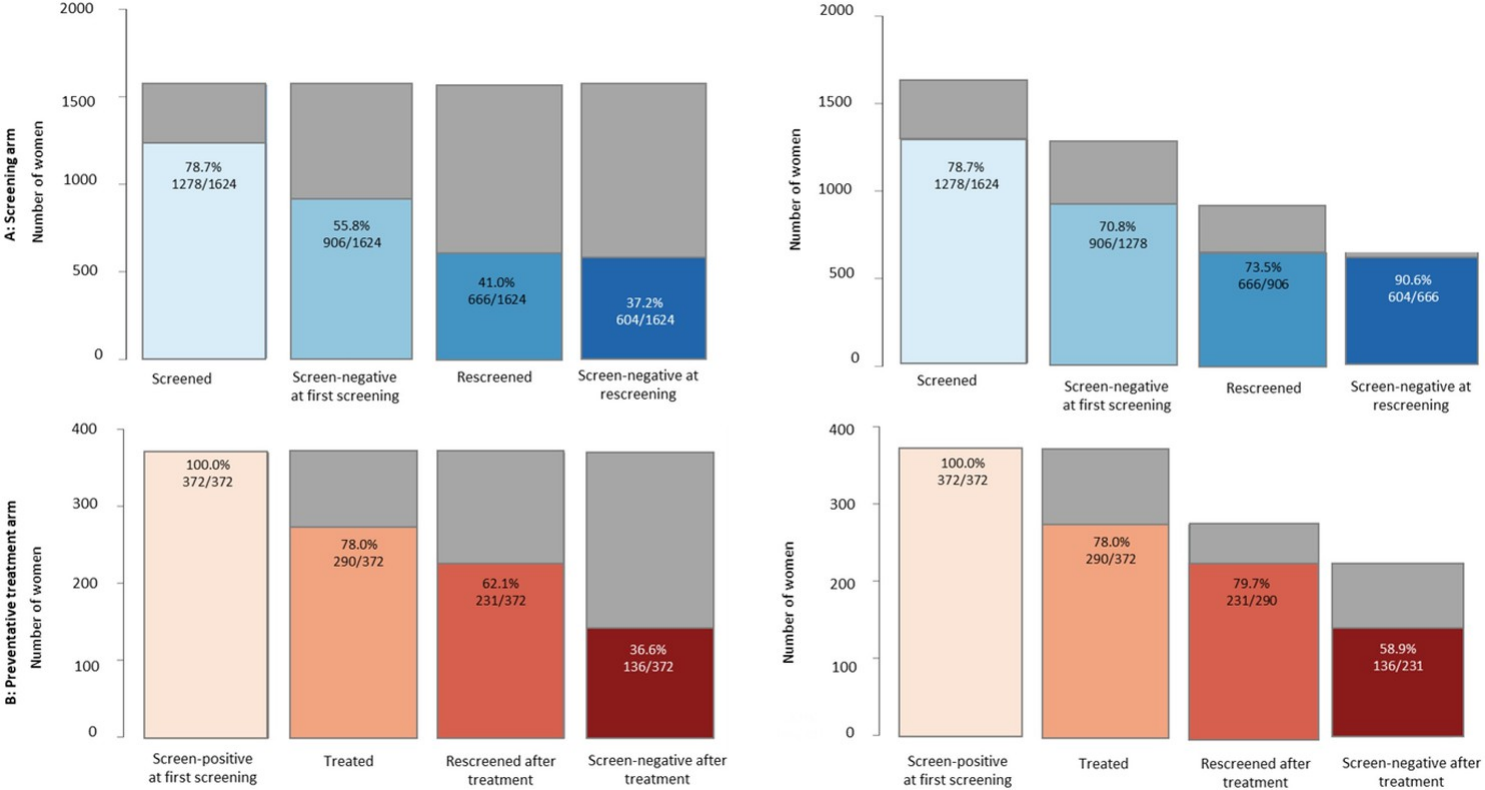
Number and percentage of women living with HIV (WLHIV) for each stage of the Cervical Cancer Screening Cascade: Screening and preventative treatment arms. Panel A: screening arm; left: percentages using a fixed denominator (denominator-denominator approach), referring to the denominator “women in care”; right: using a changing denominator (numerator-denominator approach), referring to all WLHIV reaching the previous stage; panel B: preventative treatment arm; left: percentages using a fixed denominator (denominator-denominator approach), referring to the denominator “screen-positive at first screening”; right: using a changing denominator (numerator-denominator approach), referring to all WLHIV reaching the previous stage. The guideline indicated timeframes are described in the Methods and S1 Table. Grey areas represent the percentage of eligible WLHIV who did not achieve a respective cascade stage (retention gaps).

Fig 3 shows the percentage of WLHIV reaching each stage of the cascade within guideline-indicated time-frames using fixed- (denominator-denominator) and changing-denominator (numerator-denominator) approaches, respectively. Using the changing-denominator approach, 78.7% (n = 1278/1624) of WLHIV were screened within one year of enrollment at the clinic, and 70.8% (n = 906/1278) had documented negative screening results. Among screen-negative WLHIV, 73.5% (n = 666/906) were re-screened after 12 months, and of these WLHIV, 90.7% (n = 604/666) had documented screen-negative results. Among 372 WLHIV who were screen-positive at the first screening, 78.0% (n = 290/372) were treated within 6 months. Among treated WLHIV, 79.7% (n = 231/290) were followed up within 6 months; and 58.9% (n = 136/231) had documented screen-negative results. Using a fixed-denominator, the percentage decrease from one stage to the next (Fig 2).

### Cumulative incidences along the cascade

**Fig 4:**
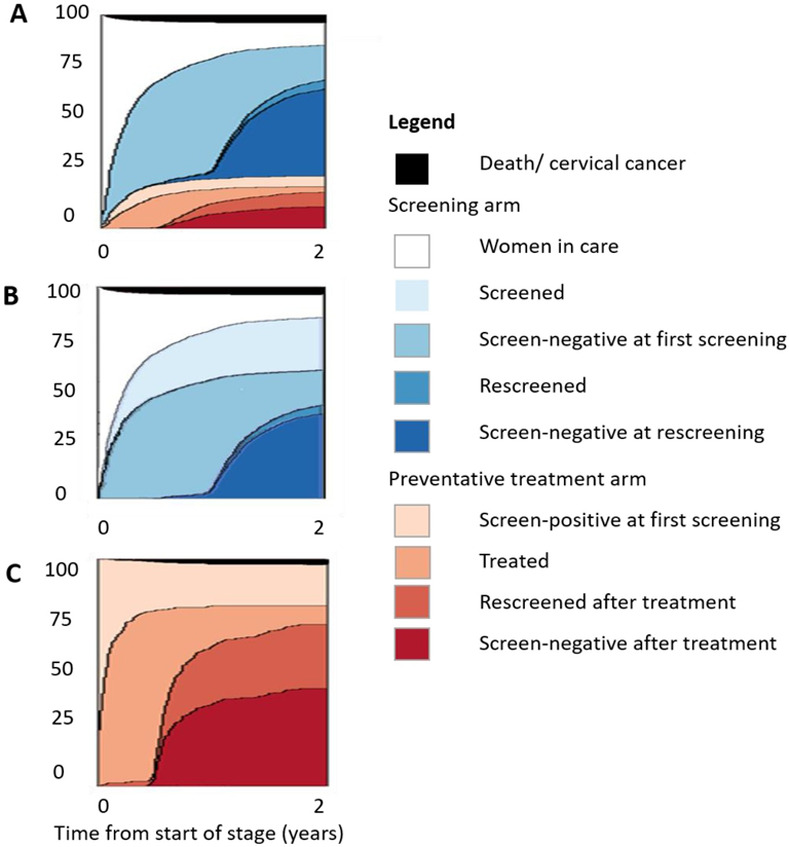
Analysis of cumulative incidence–percentage of women living with HIV (WLHIV) transitioning through the Cervical Cancer Screening Cascade by year. The two-year cumulative incidence of WLHIV transitioning to each stage of the Cervical Cancer Screening Cascade—Panel A: Cervical Cancer Screening Cascade, time zero: date of first ART clinic visit; Panel B: screening arm, time zero: date of first ART clinic visit; Panel C: preventative treatment arm, time zero: date of initial screen-positivity.

Total observation time was 1483 person-years (median 389 days, IQR 0–1959 days). During the study period, 1385 WLHIV were screened at least once (S3 Table), and the median time to screening from enrollment was 63 days (IQR 28–146). Fig 4 shows the cumulative incidence of reaching each stage of the cascade arms over two years. The cumulative incidence of receiving cervical screening within two years after clinic enrollment was 85.4% (95% CI 83.5–87.1). Of the screened WLHIV, 989 (71.4%) had a documented negative screening result at their first screening visit. The median time to documented negative test was 63 days (IQR 28–154), and the cumulative incidence of documented negative test within two years was 60.6% (95% CI 58.1–62.9). Among WLHIV who screened negative at the first screening, 791 (80.0%) received re-screening over the full study period. The median time from the first screening date to re-screening was 374 days (IQR 361–424), and the cumulative incidence of re-screening at two years was 44.9% (95% CI 42.3–47.3). At the time of re-screening, 713 (90.1%) remained screen-negative, and the cumulative incidence of negative re-screening was 40.6% (95% CI 38.1–43.0) at two years.

At their first screening, 396 WLHIV had documented positive screening results, and the median time to documented positive test was 64.5 days (IQR 28–133). Among WLHIV who screened positive, 316 (79.8%) were treated over the study period; from the first screen-positive test, the median time to treatment was 13 days (IQR 0–31) and the cumulative incidence of being treated within two years of a positive test was 79.5% (95% CI 75.1–83.2). Fourteen WLHIV had their treatment deferred due to pregnancy, sexually transmitted co-infection (STI) or prioritized HIV treatment and therefore exited the cascade (S1 Fig). Overall, 277 (87.7%) WLHIV were followed up after treatment, time to re-screening was 188 days (IQR 181–223), and cumulative incidence of re-screening within two years was 61.7% (95% CI 56.6–66.3). Among 277 WLHIV followed up after treatment of precancerous cervical lesions, 169 (61.0%) had documented screen-negative results and the cumulative incidence of testing negative at re-screening at two years was 36.1% (95% CI 31.2–40.7). Notably, half of the WLHIV receiving treatment received cryotherapy (n = 152/316, 48.1%), usually on the same day as screening (median time to cryotherapy: 0 days, IQR 0–15). The median time to treatment for the remaining LEEP treatments was 26.5 days (IQR 9–58). Similarly, among WLHIV who tested negative at the post-treatment re-screening, 56.2% (n = 95/169) had been treated with cryotherapy and 43.8% (n = 74/169) with LEEP (S2 Fig). During the study period, 117 WLHIV died of any cause; median time to death was 133 days after enrollment (IQR 52–421).

### Cancer diagnoses

Fifteen WLHIV were diagnosed with cervical cancer and were referred for treatment during the study period; the median time from enrolment into the ART clinic to cancer diagnosis was 24 days (IQR 0–239). Three WLHIV received subsequent radical hysterectomy, five received combination chemo-radiotherapy, one radiotherapy, and one chemotherapy. Treatment of the remaining five WLHIV was unknown. By the end of the study period, seven of these WLHIV died, seven were alive, and one was lost to follow-up.

### Factors associated with retention

We assessed patient factors associated with retention in the cascade defined as attending re-screening in either arm (Table 2). Women were more likely to be retained between 2015–2018, compared to 2012–2015 (aHR 1.26, 95% CI 1.10–1.43). HIV RNA viral loads (>1000 vs <50 copies/mm^3^, aHR 1.33, 95% CI 1.16–1.53) and having more children (parity 1–3 vs 0, aHR 1.46, 95% CI 1.15–1.86) were also associated with retention in the cascade. There was no evidence for an association of the patient factors we assessed and having a negative re-screening test result after treatment (S4 Table).

**Table 2:** Crude and adjusted hazard ratios for factors associated with retention in the Cervical Cancer Screening Cascade.

	Univariable analyses			Multivariable analysis		
	n = 1251			n = 1251		
	HR	95% CI	P value	HR	95% CI	P value
**Age, years**						
18–25	2.02	1.61–2.54		1.40	1.07–1.82	
25–44	1	-	<0.001	1	-	0.025
>45	2.19	1.71–2.80		1.50	1.12–2.03	
**Relationship**						
Single	0.78	0.67–0.90		0.83	0.71–0.98	
Relationship	1	-	0.002	1	-	0.076
Separated	0.98	0.85–1.14		0.92	0.78–1.08	
**Employment**						
Unemployed	1	-		1	-	
Employed	0.98	0.86–1.12	<0.001	0.93	0.81–1.06	0.388
Student	0.45	0.29–0.70		0.81	0.51–1.30	
**Parity**						
0	1	-		1	-	
1 to 3	1.77	1.44–2.19	<0.001	1.46	1.15–1.86	0.008
>3	1.37	1.08–1.73		1.41	1.07–1.85	
**HIV RNA viral load at VIA screening, copies/mm** ^**3**^						
<50	1	-		1	-	
50–1000	1.21	0.99–1.47	<0.001	1.19	0.96–1.47	<0.001
>1000	1.36	1.19–1.55		1.33	1.16–1.53	
**Calendar period enrolled**						
Early years: 2012–2014	1	-		1	-	
Later years: 2015–2017	1.31	1.16–1.49	<0.001	1.26	1.10–1.43	<0.001
**Nadir CD4 cell count, cells/mm3**						
<200	1	-				
200–349	1.02	0.86–1.29				
350–500	1.40	1.86–1.26	0.977			
>500	1.02	0.84–1.24				
**HIV RNA viral load at ART enrollment, copies/mm** ^**3**^						
<50	1	-				
50–1000	0.79	0.62–1.01	0.150			
>1000	0.96	0.84–1.10				

Retention in the Cervical Cancer Screening Cascade is defined as all women living with HIV (WLHIV) achieving rescreening in either arm (screening or preventative treatment) of the Secondary Cervical Cancer Prevention Cascade. HR: hazards ratio; n: number of women; CI: confidence interval; HIV: human immunodeficiency virus; RNA: Ribonucleic acid; VIA: visual inspection with acetic acid; n: number of WLHIV.

## Discussion

We used a cascade approach to assess the full continuum of cervical screening among WLHIV enrolled in a large ART program with integrated screening services in Harare, Zimbabwe, between 2012 and 2018. This revealed important bottlenecks that need to be addressed at the clinic to improve the efficacy of the screening program. The cumulative incidence of screening at two years after the first clinic visit was 85%. However, only 80% of WLHIV received treatment and the cumulative incidence of a negative test was 36% at two years after initial screen-positivity. Women with high HIV RNA viral loads and more children were more likely to be retained in the cascade. Retention of women through all cascade steps improved over time.

We evaluated the entire Cerivcal Cancer Screening Cascade using individual patient-level data from routine care, extending from previous research [20, 21, 26–30]. An important strength is that this is a denominator-denominator linked longitudinal study, in a single, well defined population [14]. The analysis of cumulative incidence has the advantage of appropriately taking into account censoring and competing events. In our study, few WLHIV died, developed cervical cancer, or were lost to follow-up; therefore, the cumulative incidences are similar to the analysis of simple percentages.

Our study has several limitations. While Newlands Clinic is an excellent setting to test the cascade concept, the results may not apply to clinics where cervical screening is not integrated. We tested the approach on VIA and PAP-based screening program, in a centre with good capacity for data-collection. To assess scalability, the approach requires testing different screening methods and in more clinics and countries. The retention gaps between eligibility and reaching cascade stages (Fig 3, grey area) were not fully explained by data collected in our study. Data explaining reasons for reduced retention across the Cervical Cancer Screening Cascade stages may improve the longitudinal approach. We focused on a cascade for secondary prevention; however, increasing the cascade’s breadth to include vaccination and care for WLHIV diagnosed with cervical cancer would further inform cervical cancer control strategies.

Our study overcame some limitations of previous studies describing the cervical screening continuum [20, 21, 26–30]. Only one study illustrated a full cascade graphically [26]. In contrast, previous studies focused on specific stages of the cascades (e.g. initial screening results [20, 29], referrals [28, 30], treatment [20, 21]). Previous studies did not analyze the time to reaching a stage even when studies spanned several years [26, 27, 29]. Our longitudinal approach allowed appropriate time-to-event analyses and avoided truncations to standardize the period of reaching each stage. This approach offers a deeper understanding of women’s movement through the cascade and may document changes in the population and health system over time. Similar retention gaps have been identified in other studies between screening and treatment [21, 26, 28, 29], which is more pronounced when an interim triage test (e.g. colposcopy) is also required [20, 27, 28]. No other studies included re-screening measures. Furthermore, our cascade focuses on WLHIV and assessing the full continuum of care for this high-risk group offers insights into their specific screening needs and may allow more risk-stratified approaches to be developed in the future [31, 32].

The WHO posits two national screening targets for the secondary prevention of cervical cancer: 70% of WLHIV should be screened with a high-performance test from 25 years, and 90% of women who screen-positive should be treated [1]. More than 70% of WLHIV aged 18 years and above, seen at Newlands Clinic, were screened using the screening tests available over the study period; VIAC or PAP-smear. However, only 80% of those who screened positive were treated. We do not know the intended treatments for those missed. Still, because clinic guidelines adhere to a see-and-treat principle, we presume missed treatments were more likely LEEP because cryotherapy would have been applied on the same day as screening. Interventions to improve adherence to treatment for women who do not qualify for same-day cryotherapy may help the program reach the WHO target. Still, more research is needed to understand this treatment gap and to change policies accordingly. Qualitative methods may be helpful to elucidate reasons for non-attendace across the Cervical Cancer Screening Cascade stages among WLHIV.

Preventing cervical cancer depends on effective treatment of precancerous lesions. Only few women had a negative test result at post-treatment re-screening (36% at two years). This is in line with recent studies reporting high pre-cancer treatment failure among WLHIV [33, 34]. Most women in our study were screened after treatment by seven months, so it would be unlikely, though not impossible, that new disease would develop in such a short time frame. The risk of residual or recurrent cervical disease is higher in WLHIV if they are treated with cryotherapy instead of LEEP [34, 35], and also when the excision is incomplete [36, 37], but post-treatment follow-up recommendations do not vary by treatment modality. ART status and immunodeficiency may also be associated with differences in CIN treatment outcomes [31]. The relatively low screening test accuracy of VIAC may account for over-treatment and persistent false-positive tests at re-screening [38]. WithoutSTI testing and biopsies to confirm the need for treatment, it is difficult to quantify the potential misclassification of disease. Additionally, post-treatment screening tests must be delayed until an HPV infection has been suppressed and tissues repair themselves, but not delayed so long for a new HPV infection to develop new cervical neoplasia. A sentinel study of post-treatment screening test accuracy among WLHIV in Kenya found no single test was both highly sensitive and specific: VIA sensitivity was 27% and specificity 82%; HPV sensitivity was 85% and specificity 54% [35]. We have yet to determine the ideal interval for re-testing WLHIV, what test-positivity means in terms of the risk and timeline for further cervical pre-cancerous lesions and more information in also required on treatments and their long-term efficacy in WLHIV [39]. We found that women with high HIV RNA viral loads and more children were more likely to be retained in the cascade. In the Newlands clinical context, many additional services are available for high-risk patients, including adolescents, those with poor virological control, and older women with multiple comorbidities. These women may visit the clinic more frequently and therefore be more likely to receive cervical cancer screening and follow-up management.

Our study showed, that using the cascade approach to analyse the full continuum of cervical screening in one ART clinic with integrated screening services can facilitate the improvement of screening programs by identifying gaps in the provision of care. Our study used a longitudinal cohort design, however, we also illustrated the cascade using simple percentages that could be calculated using aggregate data in sites without the ability to collect longitudinal patient-level data on cervical cancer screening. This suggests that the approach could be scalable to settings with less robust patient data-systems. Our Cervical Cancer Screening Cascade could therefore be used more widely to support the WHO goal to eliminate cervical cancer. Monitoring the full continuum of cervical cancer screening provides specific information about how to improve the effectiveness of screening programs including screening results post-treatment. We found that even if the WHO targets for coverage and treatment are met, a high proportion of WLHIV remain screen positive at follow-up and may remain at high risk for cancer. We suggest future research should: (i) test the scalability of the Cascade in cervical screening comparatively across sites with and without integrated screening services and countries; (ii) consider additional methods for understanding gaps in cascade stages, including qualitative studies to understand poor retention; (iii) investigate precancer dynamics in WLHIV and understand the clinical significance of positive screening tests after treatment.

## Conclusions

Using a cascade approach to evaluate the full continuum of cervical cancer screening at Newlands clinic in Zimbabwe, we found that less-than 80% of WLHIV received treatment after screen-positive tests and less-than 40% of these women were screen-negative at follow-up. Interventions are needed to improve linkage to treatment for screen-positive women and further studies are needed to understand the clinical significance of screen-positive tests at follow-up among WLHIV. Our cascade identifies priorities for improvement along the full continuum of cervical cancer screening. These gaps in the continuum of care must be addressed in order to prevent cervical cancer. We also demonstrate that complex and simple methods for analysis can be used to evaluate the cascade, supporting its scalability. Scaled-up, the cascade approach may be useful in supporting the WHO goal to eliminate cervical cancer.

## Supporting information

S1 Fig Patient flow in cervical screening during the full study period. (TIF)

S2 Fig The preventative treatment arm, disaggregated by mode of treatment. (TIF)

S1 Text Study site details. (DOCX)

S2 Text Questionnaire of inclusivity. (DOCX)

S1 Table Definitions for conceptual model: Secondary cervical cancer prevention cascade—Screening and preventative treatment. (DOCX)

S2 Table Extraction form. (DOCX)

S3 Table Results of the cervical cancer prevention cascade using the longitudinal approach. (DOCX)

S4 Table Crude and adjusted hazard ratios for factors associated with screen-negativity after treatment. (DOCX)
