# Supplementary figures and images for: Cervical Cancer Screening Cascade for women living with HIV: A cohort study from Zimbabwe
Source: PLOS Glob Public Health. 2022 Feb 2;2(2):e0000156. doi: 10.1371/journal.pgph.0000156 (PMC9974171; doi:10.1371/journal.pgph.0000156)

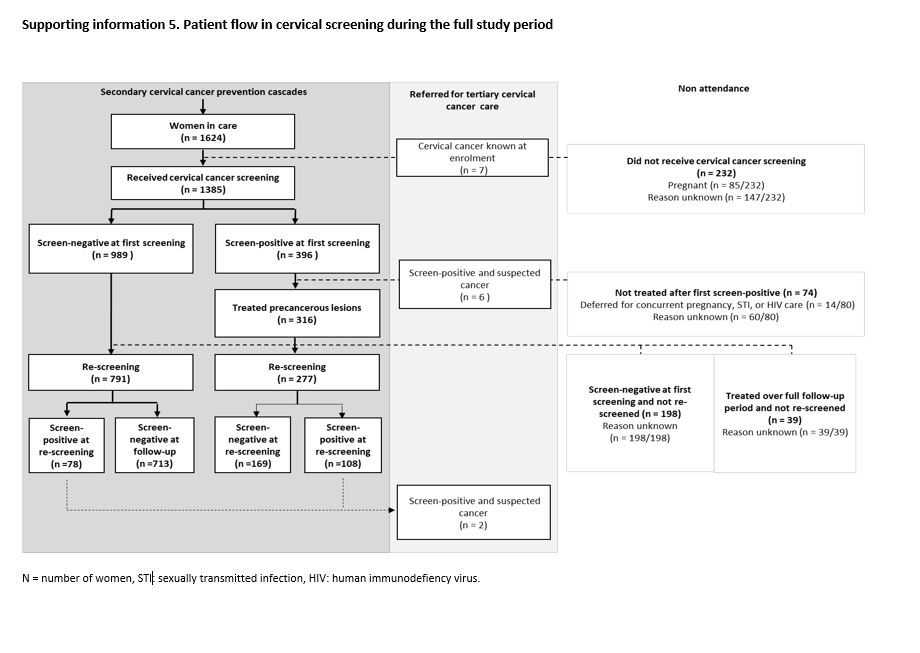

Supplement: S1 Fig — (TIF) [file pgph.0000156.s001.tif]

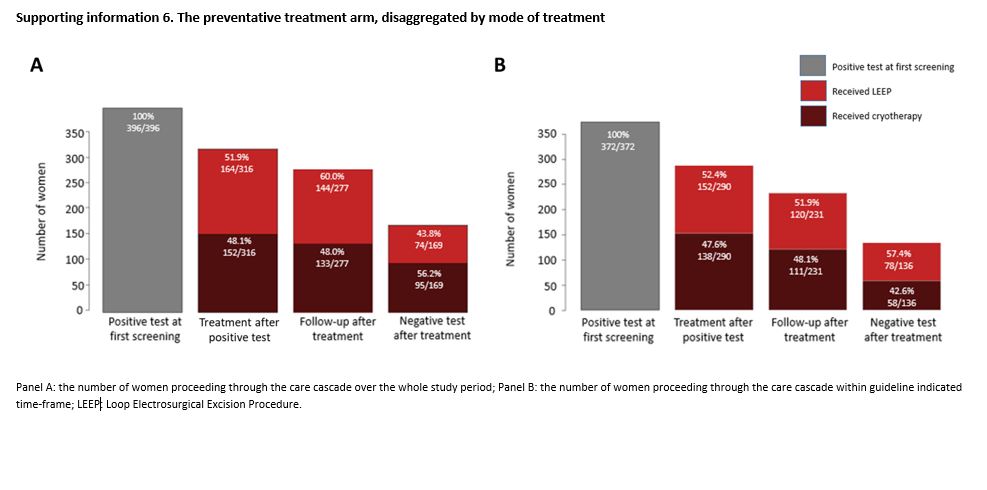

Supplement: S2 Fig — (TIF) [file pgph.0000156.s002.tif]
