# Supplementary material for: Cervical Cancer Screening Cascade for women living with HIV: A cohort study from Zimbabwe
Source: PLOS Glob Public Health. 2022 Feb 2;2(2):e0000156. doi: 10.1371/journal.pgph.0000156 (PMC9974171; doi:10.1371/journal.pgph.0000156)
Supplement: S1 Text — (DOCX) [file pgph.0000156.s003.docx]

**Supporting information 1. Study site details**

Since 2004, Newland’s Clinic has provided comprehensive healthcare services for people living with HIV. The clinic has had protocols in place to track patient response to treatment using HIV RNA viral loads supported by its own independent laboratory since 2013. The clinic also provides a wide range of additional services aiming to improve the wellbeing of their patients as well as their families. Patient nutrition is monitored, and food supplementation is provided for the family if food security cannot be guaranteed. Patients are regularly monitored for non-communicable diseases. Specialized adolescent and perinatal care with psychological supports are also available. The Clinic is based on a nurse-led model of care, with doctors supporting nurses with clinical decisions. Weekly case-based teaching for all staff, and Advanced HIV management courses for external health-care workers are offered and supported by the Ministry of Health and Child Care in Zimbabwe.

The cervical cancer screening services have been in place since January 2012. All women who are sexually active are referred to have cervical cancer screening, however this is not recorded systematically in the electronic data-base. Screening is predominantly by visual inspection with acetic acid and cervicography (VIAC). This procedure requires a vaginal speculum examination and the application of 3-5% acetic acid to the cervix. The appearance of white plaques on the surface of the cervix may indicate the presence of precancerous or cancerous disease. The cervix is examined by naked eye and images are obtained using a Cannon camera (Powershot SX40HS, 12MP CMOS sensor). Cervical images are stored in the patient notes in the clinic database, can subsequently be reviewed by a local gynecologist. If a woman is suspected of having cervical cancer, she is referred for further treatment to a tertiary center. For woman who do not have a visible transformation zone and are therefore not suitable for VIAC, a Papanicolaou (PAP) test is done. Ambiguous cervical lesions and those suspicious for cervical cancer may be biopsied. Pre-cancerous lesions of the cervix can be treated on the same day as screening by nurses who are trained in cryotherapy, if women have amenable lesions. A local gynecologist comes to the clinic weekly to provide Loop electrosurgical excision procedure (LEEP) treatment and a second opinion on cervigrams. Women with signs or symptoms of sexually transmitted infections (STIs) are offered treatment which may delay treatment of concurrent cervical lesions.
