# Supplementary material for: Cervical Cancer Screening Cascade for women living with HIV: A cohort study from Zimbabwe
Source: PLOS Glob Public Health. 2022 Feb 2;2(2):e0000156. doi: 10.1371/journal.pgph.0000156 (PMC9974171; doi:10.1371/journal.pgph.0000156)

**Supporting information 2. Definitions for conceptual model:** **Secondary cervical cancer prevention cascade:**  s**creening and preventative treatment**


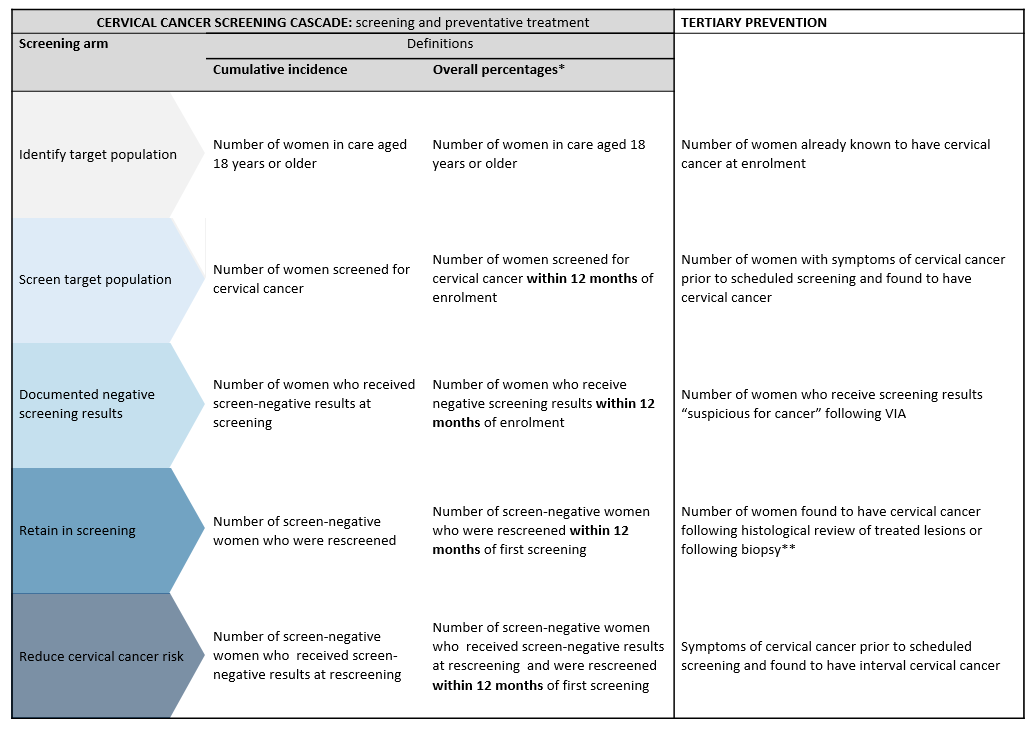


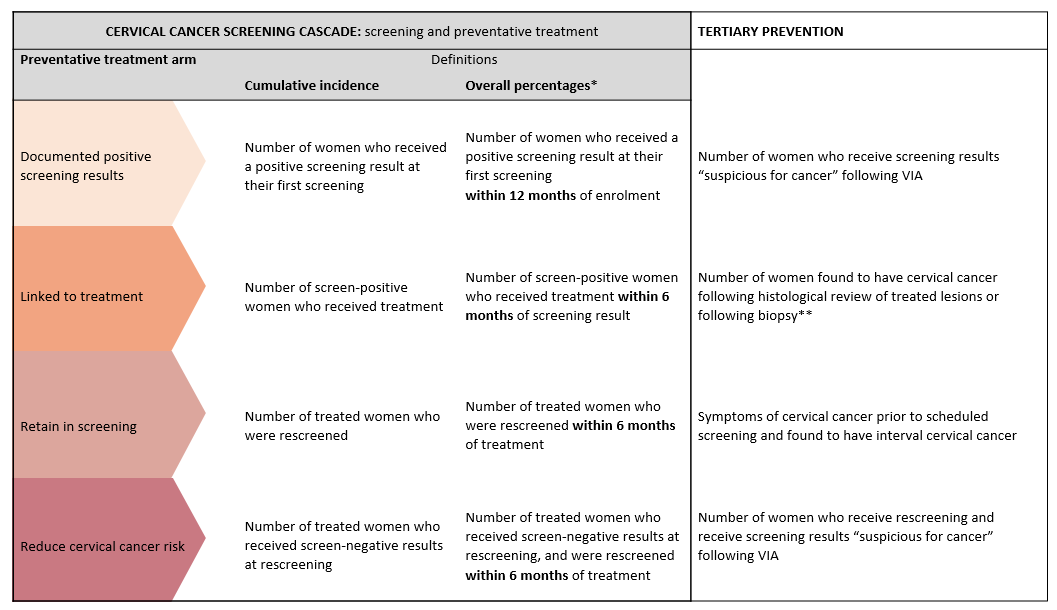

Supplement: S1 Table — (DOCX) [file pgph.0000156.s005.docx]
