# Supplementary material for: Cervical Cancer Screening Cascade for women living with HIV: A cohort study from Zimbabwe
Source: PLOS Glob Public Health. 2022 Feb 2;2(2):e0000156. doi: 10.1371/journal.pgph.0000156 (PMC9974171; doi:10.1371/journal.pgph.0000156)
Supplement: S2 Table — (DOCX) [file pgph.0000156.s006.docx]

**Supporting information 3. Extraction form**

The following extraction form was created *a-priori*. Below we indicate data collected for each stage of the cascade.

Please note that since this extraction form was developed, names of cascade stages have changed.

| **Abbreviation list** | | | |
| --- | --- | --- | --- |
| **ART** | antiretroviral therapy | **ICC** | invasive cervical cancer |
| **ASCUS** | atypical squamous cells of undetermined significance | **ID** | Identification |
| **CD4** | cluster of differentiation 4 | **IUCD** | intrauterine contraceptive device |
| **CIN** | cervical intraepithelial neoplasia | **LEEP** | Loop Electrosurgical Excision Procedure |
| **COCP** | combined oral contraceptive pill | **LLETZ** | Large Loop Excision of the Transformation Zone |
| **EUA** | Examination under Anesthetic | **STI** | sexually transmitted infections |
| **FU** | Follow-up | **VIA** | Visual Inspection with Acetic Acid |
| **HIV** | human immunodeficiency virus | **WHO** | World Health Organization |
| **HPV** | human papillomavirus |  |  |

| **Baseline site data** | | | | | |
| --- | --- | --- | --- | --- | --- |
| **Question**  **number** | **Variable name** | **Description** | **Format** | **Code** | **Notes** |
| 6 | country | In which country is your ART program located? | Categorical | 1 = Zimbabwe  2 = Zambia  3 = Malawi  4 = South Africa  5 = Mozambique  88 = other |  |
| 7 | country_c | If response to country = 88, please insert country name | String |  |  |
| 8 | site_type | Site Type  (for example, HIV clinic, Maternal and Child health clinic, Maternal and family health clinic, Sexual health clinic, antenatal clinic, clinical research site, primary care general clinic, other) | String |  |  |
| 9 | affiliations | If research site, please list research networks your site is involved in and university affiliations | String |  |  |
| 10 | site_loc | Site Location | Categorical | 1 = urban  2 = rural  3 = peri-urban |  |
| 11 | art_start | Date of onset of ART program | Date | YYYY-MM-DD  (99 for any or all missing components) |  |
| 12 | viral_loads_start | When did this clinic start measuring viral loads for monitoring HIV treatment | Date | YYYY-MM-DD  (99 for any or all missing components) |  |
| 13 | cx_screen_start | Date of onset of cervical screening program | Date | YYYY-MM-DD  (99 for any or all missing components) |  |
| 14 | data_entry_start | Years included in following survey | Date | Start date: YYYY-MM-DD  (99 for any or all missing components) |  |
| 15 | data_entry_end |  |  | End date: YYYY-MM-DD  (99 for any or all missing components) |  |
| 16 | site_level_screenby | Cervical cancer screening is done by:  (more than one answer may be appropriate) | Categorical | 1 = nurse  2 = lay health worker  3 = doctor other than gynecologist  4 = gynecologist  77 = don’t know  88 = other |  |
| 17 | site_level_screenby_c | If response to site_level_screenby = other, please describe | String |  |  |
| 18 | ref_for_cx_screen | Women are referred to your clinic for cervical cancer screening | Categorical | 0 = no  1 = yes  77 = don’t know |  |
| 19 | avial_via | VIA screening is available at this clinic | Categorical | 0 = no  1 = yes  77 = don’t know |  |
| 20 | avial_via_d | Start date: VIA has been available since | Date | YYYY-MM-DD  (99 for any or all missing components) |  |
| 21 | avail_smear | PAP smear test can be done at this clinic | Categorical | 0 = no  1 = yes  77 = don’t know |  |
| 22 | avail_smear_d | Start date: Smear test have been available since | Date | YYYY-MM-DD  (99 for any or all missing components) |  |
| 23 | avail_hpv | HPV testing can be done at this clinic | Categorical | 0 = no  1 = yes  77 = don’t know |  |
| 24 | avail_hpv_d | Start date: HPV testing has been available since | Date | YYYY-MM-DD  (99 for any or all missing components) |  |
| 25 | cervicitis_tx_prot | Diagnosis and treatment of cervicitis is based on clinical signs and symptoms | Categorical | 0 = no  1 = yes, mostly  2 = yes, always  77 = don’t know |  |
| 26 | avail_swabs | Diagnosis and treatment of cervicitis is based on cultures obtained from swabs | Categorical | 0 = no  1 = yes  77 = don’t know |  |
| 27 | avail_swabs_d | Swabs to confirm cervicitis have been available since | Date | YYYY-MM  (99 for any or all missing components) |  |
| 28 | targetgroup | Women are eligible based on age, sexual activity or both of these features | Categorical | 1 = age  2 = sexual activity  3 = both above | If age is selected, go to site_age_target1  If not go to screen_interval_n |
| 29 | site_age_target1 | Age range of women eligible for screening at your site | Categorical | Youngest  Continuous variable |  |
| 30 | site_age_target2 |  |  | Oldest  Continuous variable |  |
| 31 | identify | Eligible women can be identified by: | Categorical | 1 = HIV clinic database  2 = social security register  3= health insurance register  4 =health service register  88 = other |  |
| 32 | invite | Are women invited to participate in cervical cancer screening opportunistically, or by another more regular/systematic method | Categorical | 1 = opportunistic  77 = don’t know  88 = other |  |
| 33 | invite_c | If a method other than Opportunistic invitation is used, please describe it | String |  |  |
| 34 | invite_method | Methods used to invite women  (select all applicable) | Categorical | 1 = letter  2 = phone call  3 = verbally at clinic  4= following campaign drives  77 = Don’t know  88= other |  |
| 35 | data_stored | Screening data is stored  (select all applicable) | Categorical | 1 = electronic,  2 = paper-based,  77 = don’t know,  88 = other |  |
| 36 | data_stored_c | If screening is stored by another means please describe | String |  |  |
| 37 | data_stored_invite | Do you capture data on whether a woman is invited for cervical screening or not?  (for example, is this recorded as text or on the database as a specific variable for each patient) | Categorical | 0 = no  1 = yes  77 = don’t know |  |
| 38 | screen_interval_n | Recommended screening interval in clinic when previous smear normal  (Please indicate in YEARS or MONTHS) | String |  |  |
| 39 | screen_interval_tx | Recommended screening interval in clinic when previous treatment for precancerous lesions  (Please indicate in YEARS or MONTHS) | String |  |  |
| 40 | price_ screen | What is the price that each women pays out of their own pocket for an initial screening visit at your clinic? (please include the currency) | String (currency) |  |  |
| 41 | cryo_tx | Can women receive cryotherapy treatment at your clinic | Categorical | 0 = no  1 = yes  77 = don’t know |  |
| 42 | leep_tx | Can women receive LEEP treatment at your clinic | Categorical | 0 = no  1 = yes  77 = don’t know |  |
| 43 | price_cin_tx | What is the price that each woman pays out of pocket for cryotherapy treatment? (please include the currency) | String (currency) |  |  |
| 44 | cancer_tx | Which cancer treatments are offered by your clinic?  (select all applicable) | Categorical | 0 = none  1 = Surgery  2 = Chemotherapy  3 = Radiotherapy  4 = hospital-based palliation  5 = home-based palliation  77 = don’t know |  |
| 45 | ref_cancer_tx | Which cancer treatments are available to patients by referral?  (select all applicable) | Categorical | 0 = none  1 = Surgery  2 = Chemotherapy  3 = Radiotherapy  4 = hospital-based palliation  5 = home-based palliation  77 = don’t know |  |
| 46 | price_cancer_tx | Please explain how cancer treatment is funded describing both Government contributions and the price women must pay out of pocket? | String |  |  |

| **Patient level screening data for women in ART programs**  Please include data on **all women, 18+ years of age, entering HIV care** between the start and end dates of your survey. | | | | | |
| --- | --- | --- | --- | --- | --- |
| **Cascade step 1: Identification**  **Baseline patient data** | | | | | |
| **Nr** | **Variable name** | **Description** | **Format** | **Code** | **Notes** |
| 100 | pid | Patient ID: Code to identify patient (Cohort Patient ID) | Numeric | Continuous variable  99 = not recorded | **This data pertains to ALL WOMEN, 18 YEARs OF AGE AND OLDER, ENTERING HIV CARE over the FULL period** of the study |
| 101 | center | Clinic code: Code for Clinic/Centre/Hospital where patient is seen. Needs to be unique within each region. | String |  |  |
| 102 | nat_n | Country of Origin/ nationality | Categorical | 1 = Zimbabwean  2 = Zambian  3 = Malawian  4 = South African  5 = Mozambican  88 = other  99 = not recorded |  |
| 103 | nat_c |  | String | If response to nat_n = 88, state nationality |  |
| 104 | bd | Birth date of woman | Date | YYYY-MM-DD  (99 for any or all missing components) |  |
| 105 | ed | Education level at time of enrollment into ART program | Categorical | 0 = none  1 = primary  2 = high school  3 = university/ college  99 = not recorded |  |
| 106 | marital | Marital status at time of enrollment into ART | Categorical | 1 = single  2 = married  3 = widowed  4 = divorced  5 = partnership  99 = not recorded |  |
| 107 | s_active | Woman is sexually active at enrollment into ART program | Categorical | 0 = no  1 = yes  99 = not recorded |  |
| 108 | pregnant | Woman is sexually active at enrollment into ART program | Categorical | 0 = no  1 = yes  99 = not recorded |  |
| 109 | employ | Employment status at time of enrollment into ART (tick all relevant) | Categorical | 0 = unemployed  1 = employed  2 = self-employed  3 = student  99 = not recorded |  |
| 110 | smoke | Cigarette smoking status at time of enrollment into ART | Categorical | 0 = never smoked  1 = current smoker  2 = ever smoked  99 = not recorded |  |
| 111 | gravid | Gravidity (the number of times that a woman has been pregnant ) at time of enrollment into ART | Numeric | Continuous variable  99 = not recorded |  |
| 112 | parity | Parity (the number of times that she has given birth to a fetus with a gestational age of 24 weeks or more, regardless of whether the child was born alive or was stillborn) at time of enrollment into ART | Numeric | Continuous variable  99 = not recorded |  |
| 113 | contraception | Contraception at time of enrollment into ART | Categorical | 0 = none  1 = combined oral contraceptive pill (COCP)  2 = intrauterine contraceptive device (IUCD)  3 = Depo-Provera (progestin injection received 3 monthly)  4 = implant (small subdermal progestogen rods – usually effective for 3 -5 years)  5 = condoms  88 = other  99 = not recorded |  |
| 114 | contraception_c | If response to contraception = 88, describe | String |  |  |
| 115 | nadir_cd4 | Nadir cd4 from clinic records | Numeric (cells/µl ) | Continuous variable  99 = not recorded |  |
| 116 | nadir_cd4_d | Date Nadir cd4 obtained | Date | YYYY-MM-DD  (99 for any or all missing components) |  |
| 117 | vl_art | Viral load at time of enrollment into ART program, (closest to enrollment) | Numeric (copies/ml) | Continuous variable  99 = not recorded |  |
| 118 | vl_art_d | Date viral load at enrolment into ART obtained | Date | YYYY-MM-DD  (99 for any or all missing components) |  |
| 119 | vl_via | Viral load at time of first VIA | Numeric | Continuous variable  99 = not recorded |  |
| 120 | vl_via_d | Date viral load at time of first VIA obtained | Date | YYYY-MM-DD  (99 for any or all missing components) |  |
| 121 | hist_sti | Previous STI reported verbally | Categorical | 0 = no  1 = yes  99 = not recorded |  |
| 122 | hist_sti_d | Date of hist_sti | Date | YYYY-MM-DD  (99 for any or all missing components) |  |
| 123 | swabs1 | Swabs done at time of first VIA | Categorical | 0 = no  1 = yes  99 = not recorded |  |
| 124 | swabs1_n | STIs found on clinically obtained swabs at time of first VIA  (select all relevant) | Categorical | 0 = no growth  1 = trichomonas  2 = chlamydia  3 = candida  4 = Gonorrhea  5 = HSV  6 = indeterminate results  88 = other  99 = not recorded |  |
| 125 | swabs1_d | Date swabs obtained | Date | YYYY-MM-DD  (99 for any or all missing components) |  |
| 126 | swabs1_c | If response to Swabs1_n = 88, describe | String |  |  |
| 127 | swabs2 | Swabs done subsequently to swabs1_d | Categorical | 0 = no  1 = yes  99 = not recorded |  |
| 128 | swabs2_n | STIs found on clinically obtained swabs at time of second VIA  (select all relevant) | Categorical | 0 = no growth  1 = trichomonas  2 = chlamydia  3 = candida  4 = Gonorrhea  5 = HSV  6 = indeterminate results  8 = normal  88 = other  99 = not recorded |  |
| 129 | swabs2-d | Date swabs obtained | Date | YYYY-MM-DD  (99 for any or all missing components) |  |
| 130 | swabs2_c | If response to Swabs2_n = 88, describe | String |  |  |
| 131 | swabs3 | Swabs done subsequently to swabs2_d | Categorical | 0 = no  1 = yes  99 = not recorded |  |
| 132 | swabs3_n | STIs found on clinically obtained swabs at time of first VIA  (select all relevant) | Categorical | 0 = no growth  1 = trichomonas  2 = chlamydia  3 = candida  4 = Gonorrhea  5 = HSV  6 = indeterminate results  88 = other  99 = not recorded |  |
| 133 | swabs3-d | Date swabs obtained | Date | YYYY-MM-DD  (99 for any or all missing components) |  |
| 134 | swabs3_c | If response to Swabs3_n = 88, describe | String |  |  |
| 135 | swabs4 | Swabs done subsequently to swabs3_d | Categorical | 0 = no  1 = yes  99 = not recorded |  |
| 136 | swabs4_n | STIs found on clinically obtained swabs at time of first VIA  (select all relevant) | Categorical | 0 = no growth  1 = trichomonas  2 = chlamydia  3 = candida  4 = Gonorrhea  5 = HSV  6 = indeterminate results  88 = other  99 = not recorded |  |
| 137 | swabs4-d | Date swabs obtained | Date | YYYY-MM-DD  (99 for any or all missing components) |  |
| 138 | swabs4_c | If response to Swabs3_n = 88, describe | String |  |  |
| 139 | art_tx_enrol | On ART treatment at the time of enrollment to HIV clinic | Categorical | 0 = no  1 = yes  99 = not recorded |  |
| 140 | art_tx_preenrol | Started ART treatment prior to enrollment in HIV clinic | Categorical | 0 = no  1 = yes  99 = not recorded |  |
| 141 | stage_bl | WHO HIV/AIDS Stage at entry into HIV care (baseline) | Categorical | 1 = 1  2 = 2  3 = 3  4 = 4  99 = not recorded |  |
| 142 | stage_bl_d | Date HIV/AIDS Stage at entry into care was assessed | Date | YYYY-MM-DD  (99 for any or all missing components) |  |
| 143 | art_d1 | Date 1^st^ line ART was started | Date | YYYY-MM-DD  (99 for any or all missing components) |  |
| 144 | art_d2 | Date 2^nd^ line ART was started | Date | YYYY-MM-DD  (99 for any or all missing components) |  |
| 145 | art_d3 | Date 3^rd^ line ART was started | Date | YYYY-MM-DD  (99 for any or all missing components) |  |
| 146 | vital | Last vital status | Categorical | 1 = alive  2 = dead  3 = lost to follow-up  99 = not recorded |  |
| 147 | cause_mort | If response to vital = 2, what was the cause of mortality | String |  |  |
| 148 | vital_d | Date that last vital status obtained  (this should be as close to data_entry_end as possible) | Date | YYYY-MM-DD  (99 for any or all missing components) |  |

| **Cascade Step 2: Invited** | | | | |  |
| --- | --- | --- | --- | --- | --- |
| 200 | invited | Woman was INVITED for screening | Numeric | 1 = yes  2 = no  99 = not recorded |  |
| 201 | invited_d | Date that woman was invited for screening | Date | YYYY-MM-DD |  |

| **Cascade Step: 3 Screened** | | | | |  |
| --- | --- | --- | --- | --- | --- |
| 300 | screened | Did the woman receive cervical cancer screening | Categorical | 0 = no  1 = yes  99 = not recorded | **If response to screened = 0, go to cascade step 4** |
| 301 | screen_via | Did the woman receive VIA screening | Categorical | 0 = no  1 = yes  99 = not recorded | **If response screen_via = 0, go to screen_pap** |
| 302 | via_1 | VIA result visit 1 | Categorical | 0 = negative  1 = positive  2 = indeterminate  3 = suspicious of cancer  99 = not recorded |  |
| 303 | via_1d | Date via_1 obtained | Date | YYYY-MM-DD  (99 for any or all missing components) |  |
| 304 | screen_pap | Did the woman receive PAP smear | Categorical | 0 = no  1 = yes  99 = not recorded | **If response to screen_pap = 0 , go to hpv_1** |
| 305 | smear_cin_1 | Smear results at visit 1, presence of neoplasia | Categorical | 0 = No neoplasia +/- benign  disease  1 = CIN1  2 = CIN2  3 = CIN3  4 = ICC  5 = ASCUS  99 = not recorded |  |
| 306 | smear_benign_1 | Smear results at visit 1, presence of benign disease  (select all relevant) | Categorical | 0 = No benign disease +/- CIN  1 = HPV  2 = cervicitis  88 = other  99 = not recorded |  |
| 307 | smear_1c | If response to smear_benign_1 = 88, explain smear (cytological) findings | String |  |  |
| 308 | smear_1d | Date Smear_1 obtained | Date | YYYY-MM-DD  (99 for any or all missing components) |  |
| 309 | screen_hpv | Woman received HPV smear | Categorical | 0 = no  1 = yes  99 = not recorded |  |
| 310 | hpv_res_1 | Result of HPV test at visit 1 | Categorical | 0 = negative  1 = positive  99 = not recorded | **If response to hpv_res_1 = 0 , go to referral_screen1** |
| 311 | hpv_1c | If response to HPV_1 = 1, please indicate the HPV sub-type (s) identified | Numerical | Continuous variable  99 = not recorded |  |
| 312 | referral_screen1 | Referral for second opinion VIA  (both within clinic and to another clinic) | Categorical | 0 = no  1 = yes  66 = unclear | **If response to referral_screen1= 0, go to referral_treat1** |
| 313 | referral_screen1d | Date referral_screen1 obtained | Date | YYYY-MM-DD  99 for any or all missing components) |  |
| 314 | referral_treat1 | Referral for treatment  (both within clinic and to another clinic) | Categorical | 0 = no  1 = yes  99 = not recorded | **If response to referral_treat1= 0, go to via_2** |
| 315 | referred_treat1d | Date referral_treat1 obtained | Date | YYYY-MM-DD  99 for any or all missing components) |  |
| 316 | via_2 | VIA result visit 2 (by same clinician or another clinician within same clinic OR at another clinic if second opinion required following initial VIA screening) | Categorical | 0 = did not receive VIA_2  1 = positive  2 = negative  3 = indeterminate  4 = suspicious of cancer  99 = not recorded | **If response to via_2 = 0, go to cascade step 4** |
| 317 | via_2d | Date VIA_2 result obtained | Date | YYYY-MM-DD  99 for any or all missing components) |  |
| 318 | via_2_c | Reason for via_2 | String | What was the reason for a second VIA (for example, cervicitis at original visit follow-up following treatment, another opinion, biopsy) |  |

| **Cascade Step 4: Diagnosed** | | | | | |
| --- | --- | --- | --- | --- | --- |
| 400 | dx_bx | Woman received diagnostic biopsy | Categorical | 0 = no  1 = yes  99 = not recorded | **If response to dx_bx = 0, go to cascade step 5** |
| 401 | bx_d | Date biopsy obtained | Date | YYYY-MM-DD  99 for any or all missing components) |  |
| 402 | bx_cin | Biopsy result at via_1 or via_2, presence of neoplasia | Categorical | 0 = No neoplasia  1 = CIN1  2 = CIN2  3 = CIN3  4 = ICC  5 = ASCUS  99 = not recorded |  |
| 403 | bx_benign | Biopsy results at via_1 or via_2, presence of benign disease  (select all relevant) | Categorical | 0 = No benign disease  1 = HPV  2 = cervicitis  88 = other  99 = not recorded |  |
| 404 | bx_c | If response to bx_benign_2= 88, describe histology findings | String |  |  |

| **Cascade Step 5: Treated** | | | | | |
| --- | --- | --- | --- | --- | --- |
| 500 | treated | Woman received treatment for cervical cancer screening | Categorical | 0 = no  1 = yes  99 = not recorded | If response to Treated = 0, go to cascade step 6 |
| 501 | cryo_1 | Received cryotherapy | Categorical | 0 = no  1 = yes  99 = not recorded |  |
| 502 | cryo_1d | Date that cryotherapy was received | Date | YYYY-MM-DD  99 for any or all missing components) |  |
| 503 | leep_1 | LEEP done | Categorical | 0 = no  1 = yes  99 = not recorded |  |
| 504 | leep_1d | Date of LEEP | Date | YYYY-MM-DD |  |
| 505 | leep_cin_1 | Histology results from leep_1, presence of neoplasia | Categorical | 0 = No neoplasia  1 = CIN1  2 = CIN2  3 = CIN3  4 = ICC  5 = ASCUS  99 = not recorded |  |
| 506 | leep_benign_1 | Histology results from leep_1, presence of benign disease  (select all relevant) | Categorical | 0 = No benign disease  1 = HPV  2 = cervicitis  88 = other  99 = not recorded |  |
| 507 | leep_1c | If response to leep_benign_1= 88, please describe histological findings | String |  |  |

| **Cascade Step 6: Follow-up 1** | | | | | |
| --- | --- | --- | --- | --- | --- |
| 600 | fu | Did woman receive first follow-up visit (fu1) | Categorical | 0 = no  1 = yes  99 = not recorded | **If response to fu = 0, go to cascade step 7 “Cancer”** |
| 601 | screenby_fu1 | Person performing first follow-up | Categorical | 1 = nurse  2 = lay health worker  3 = doctor other than gynecologist  4 = gynecologist  99 = not recorded |  |
| 602 | via_fu1 | Did the woman receive VIA at her first follow-up visit | Categorical | 0 = no  1 = yes  99 = not recorded | **If response = 0, go to smear_fu1** |
| 603 | via_res_fu1 | VIA result at fu1 | Categorical | 0 = negative  1 = positive  2 = indeterminate  3 = suspicious of cancer  99 = not recorded | **In treated women, _fu1 will inform the cascade step “treatment outcomes”**  **In all other women it will inform the cascade step “re-examine”**  **If response via_res_fu1 = 0, go to follow-up 2** |
| 604 | via_fu1_d | Date via_res_fu1 obtained | Date | YYYY-MM-DD  (99 for any or all missing components – Y/M/D) |  |
| 605 | smear_fu1 | Did the woman receive a smear at fu1 | Categorical | 0 = no  1 = yes  99 = not recorded | **If response to Smear_fu1 = 0, go to HPV_fu1** |
| 606 | smear_cin_fu1 | Smear results at fu1, presence of neoplasia | Categorical | 0 = No neoplasia +/- benign disease  1 = CIN1  2 = CIN2  3 = CIN3  4 = ICC  5 = ASCUS  99 = not recorded |  |
| 607 | smear_benign_fu1 | Smear results at fu1, presence of benign disease  (select all relevant) | Categorical | 0 = No benign disease +/- CIN  1 = HPV  2 = cervicitis  88 = other  99 = not recorded |  |
| 608 | smear_fu1_c | If response to smear_benign_fu1 = 88, describe smear (cytological) findings | String |  |  |
| 609 | smear_fu1_d | Date of smear obtained at fu1 | Date | YYYY-MM-DD  (99 for any or all missing components – Y/M/D) |  |
| 610 | hpv_ fu1 | Woman received HPV testing at follow-up | Categorical | 0 = no  1 = yes  99 = not recorded | **If response hpv_ fu1 = 0, go to bx_fu1** |
| 611 | hpv_res_ fu1 | Result of HPV test at fu1 | Categorical | 0 = negative  1 = positive  99 = not recorded |  |
| 612 | hpv_fu1_c | If response to hpv_ fu1 = 1, please indicate the HPV sub-type identified | Numerical | Continuous variable  99 = not reported |  |
| 613 | bx_fu1 | Was a biopsy done at fu1 | Categorical | 0 = no  1 = yes  99 = not recorded | **If response to**  bx_fu1**= 0, go to** cryo_fu1 |
| 614 | bx_fu1_d | Date that the biopsy from fu1 was obtained | Date | YYYY-MM-DD  (99 for any or all missing components – Y/M/D) |  |
| 615 | bx_cin_fu1 | Biopsy result at fu1, presence of neoplasia | Categorical | 0 = No neoplasia  1 = CIN1  2 = CIN2  3 = CIN3  4 = ICC  5 = ASCUS  99 = not recorded |  |
| 616 | bx_benign_fu1 | Smear results at fu1, presence of benign disease  (select all relevant) | Categorical | 0 = No benign disease  1 = HPV  2 = cervicitis  88 = other  99 = not recorded |  |
| 617 | bx_fu1_c | If response to bx_benign_fu1 = 88, explain the histological findings | String |  |  |
| 618 | treat_fu1 | Did the woman receive treatment at fu1 | Categorical | 0 = no  1 = yes  99 = not recorded |  |
| 619 | cryo_fu1 | Received cryotherapy at fu1 | Categorical | 0 = no  1 = yes  99 = not recorded | **If response to cryo_fu1 = 0, go to leep_fu1** |
| 620 | cryo_fu1_d | Date that Cryo_fu1 was received | Date | YYYY-MM-DD  (99 for any or all missing components – Y/M/D) |  |
| 621 | leep_fu1 | LEEP done at fu1 | Categorical | 0 = no  1 = yes  99 = not recorded | **If response to leep_fu1 = 0, go ref_fu1** |
| 622 | leep_fu1_d | Date that leep_fu1 was performed | Date | YYYY-MM-DD  (99 for any or all missing components – Y/M/D) |  |
| 623 | leep_cin_fu1 | Histology results from leep_fu1, presence of neoplasia | Categorical | 0 = No neoplasia  1 = CIN1  2 = CIN2  3 = CIN3  4 = ICC  5 = ASCUS  99 = not recorded |  |
| 624 | leep_benign_fu1 | Histology results from leep_fu1, presence of benign disease  (select all relevant) | Categorical | 0 = No benign disease  1 = HPV  2 = cervicitis  88 = other  99 = not recorded |  |
| 625 | leep_fu1_c | If response to leep_benign_fu1 = 88, please describe (histological) findings | String |  |  |
| 626 | ref_fu1 | Referral made at fu1 | Categorical | 0 = no referral made  1 = referral for further screening  2 = referral for treatment pre-cancer  3 = referral for treatment of suspected cancer  88 = other  99 = not recorded | **If response to ref_fu1 = 0, 1, 2, 88, 99, go to follow-up 2**  **If response to ref_fu1 = 3, go to Cascade step 8** |
| 627 | ref_fu1c | If response to ref_fu1 = 88, please explain | String |  |  |
| 628 | ref_date_fu1 | Date referral at follow-up 1 obtained | Date | YYYY-MM-DD  (99 for any or all missing components – Y/M/D) |  |

| **Follow-up2** | | | | | |
| --- | --- | --- | --- | --- | --- |
| 700 | fu | Did woman receive second follow-up visit (fu2) | Categorical | 0 = no  1 = yes  99 = not recorded | **If response to fu = 0, go to cascade step 7 “Cancer”** |
| 701 | screenby_fu2 | Person performing second follow-up | Categorical | 1 = nurse  2 = lay health worker  3 = doctor other than gynecologist  4 = gynecologist  99 = not recorded |  |
| 702 | via_fu2 | Did the woman receive VIA at her second follow-up visit | Categorical | 0 = no  1 = yes  99 = not recorded | **If response = 0, go to smear_fu2** |
| 703 | via_res_fu2 | VIA result at fu2 | Categorical | 0 = negative  1 = positive  2 = indeterminate  3 = suspicious of cancer  99 = not recorded | **In treated women, _fu2 will inform the cascade step “treatment outcomes”**  **In all other women it will inform the cascade step “re-examine”**  **If response via_res_fu2 = 0, go to follow-up 2** |
| 704 | via_fu2_d | Date via_res_fu2 obtained | Date | YYYY-MM-DD  (99 for any or all missing components – Y/M/D) |  |
| 705 | smear_fu2 | Did the woman receive a smear at fu2 | Categorical | 0 = no  1 = yes  99 = not recorded | **If response to Smear_fu2 = 0, go to HPV_fu2** |
| 706 | smear_cin_fu2 | Smear results at fu2, presence of neoplasia | Categorical | 0 = No neoplasia +/- benign disease  1 = CIN1  2 = CIN2  3 = CIN3  4 = ICC  5 = ASCUS  99 = not recorded |  |
| 707 | smear_benign_fu2 | Smear results at fu2, presence of benign disease  (select all relevant) | Categorical | 0 = No benign disease +/- CIN  1 = HPV  2 = cervicitis  88 = other  99 = not recorded |  |
| 708 | smear_fu2_c | If response to smear_benign_fu2 = 88, describe smear (cytological) findings | String |  |  |
| 709 | smear_fu2_d | Date of smear obtained at fu2 | Date | YYYY-MM-DD  (99 for any or all missing components – Y/M/D) |  |
| 710 | hpv_ fu2 | Woman received HPV testing at follow-up | Categorical | 0 = no  1 = yes  99 = not recorded | **If response hpv_ fu2 = 0, go to bx_fu2** |
| 711 | hpv_res_ fu2 | Result of HPV test at fu2 | Categorical | 0 = negative  1 = positive  99 = not recorded |  |
| 712 | hpv_fu2_c | If response to hpv_ fu2 = 1, please indicate the HPV sub-type identified | Numerical | Continuous variable  99 = not reported |  |
| 713 | bx_fu2 | Was a biopsy done at fu2 | Categorical | 0 = no  1 = yes  99 = not recorded | **If response to**  bx_fu2**= 0, go to** cryo_fu2 |
| 714 | bx_fu2_d | Date that the biopsy from fu2 was obtained | Date | YYYY-MM-DD  (99 for any or all missing components – Y/M/D) |  |
| 715 | bx_cin_fu2 | Biopsy result at fu2, presence of neoplasia | Categorical | 0 = No neoplasia  1 = CIN1  2 = CIN2  3 = CIN3  4 = ICC  5 = ASCUS  99 = not recorded |  |
| 716 | bx_benign_fu2 | Smear results at fu2, presence of benign disease  (select all relevant) | Categorical | 0 = No benign disease  1 = HPV  2 = cervicitis  88 = other  99 = not recorded |  |
| 717 | bx_fu2_c | If response to bx_benign_fu2 = 88, explain the histological findings | String |  |  |
| 718 | treat_fu2 | Did the woman receive treatment at fu2 | Categorical | 0 = no  1 = yes  99 = not recorded |  |
| 719 | cryo_fu2 | Received cryotherapy at fu2 | Categorical | 0 = no  1 = yes  99 = not recorded | **If response to cryo_fu2 = 0, go to leep_fu2** |
| 720 | cryo_fu2_d | Date that Cryo_fu2 was received | Date | YYYY-MM-DD  (99 for any or all missing components – Y/M/D) |  |
| 721 | leep_fu2 | LEEP done at fu2 | Categorical | 0 = no  1 = yes  99 = not recorded | **If response to leep_fu2 = 0, go ref_fu2** |
| 722 | leep_fu2_d | Date that leep_fu2 was performed | Date | YYYY-MM-DD  (99 for any or all missing components – Y/M/D) |  |
| 723 | leep_cin_fu2 | Histology results from leep_fu2, presence of neoplasia | Categorical | 0 = No neoplasia  1 = CIN1  2 = CIN2  3 = CIN3  4 = ICC  5 = ASCUS  99 = not recorded |  |
| 724 | leep_benign_fu2 | Histology results from leep_fu2, presence of benign disease  (select all relevant) | Categorical | 0 = No benign disease  1 = HPV  2 = cervicitis  88 = other  99 = not recorded |  |
| 725 | leep_fu2_c | If response to leep_benign_fu2 = 88, please describe (histological) findings | String |  |  |
| 726 | ref_fu2 | Referral made at fu2 | Categorical | 0 = no referral made  1 = referral for further screening  2 = referral for treatment precancer  3 = referral for treatment of suspected cancer  88 = other  99 = not recorded | **If response to ref_fu2 = 0, 1, 2, 88, 99, go to follow-up 2**  **If response to ref_fu2 = 3, go to Cascade step 8** |
| 727 | ref_fu2c | If response to ref_fu2 = 88, please explain | String |  |  |
| 728 | ref_date_fu2 | Date referral at follow-up 1 obtained | Date | YYYY-MM-DD  (99 for any or all missing components – Y/M/D) |  |

| **Follow-up3** | | | | | |
| --- | --- | --- | --- | --- | --- |
| 800 | fu | Did woman receive third follow-up visit (fu3) | Categorical | 0 = no  1 = yes  99 = not recorded | **If response to fu = 0, go to cascade step 7 “Cancer”** |
| 801 | screenby_fu3 | Person performing third follow-up | Categorical | 1 = nurse  2 = lay health worker  3 = doctor other than gynecologist  4 = gynecologist  99 = not recorded |  |
| 802 | via_fu3 | Did the woman receive VIA at her third follow-up visit | Categorical | 0 = no  1 = yes  99 = not recorded | **If response = 0, go to smear_fu3** |
| 803 | via_res_fu3 | VIA result at fu3 | Categorical | 0 = negative  1 = positive  2 = indeterminate  3 = suspicious of cancer  99 = not recorded | **In treated women, _fu3 will inform the cascade step “treatment outcomes”**  **In all other women it will inform the cascade step “re-examine”**  **If response via_res_fu3 = 0, go to follow-up 2** |
| 804 | via_fu3_d | Date via_res_fu3 obtained | Date | YYYY-MM-DD  (99 for any or all missing components – Y/M/D) |  |
| 805 | smear_fu3 | Did the woman receive a smear at fu3 | Categorical | 0 = no  1 = yes  99 = not recorded | **If response to Smear_fu3 = 0, go to HPV_fu3** |
| 806 | smear_cin_fu3 | Smear results at fu3, presence of neoplasia | Categorical | 0 = No neoplasia +/- benign disease  1 = CIN1  2 = CIN2  3 = CIN3  4 = ICC  5 = ASCUS  99 = not recorded |  |
| 807 | smear_benign_fu3 | Smear results at fu3, presence of benign disease  (select all relevant) | Categorical | 0 = No benign disease +/- CIN  1 = HPV  2 = cervicitis  88 = other  99 = not recorded |  |
| 808 | smear_fu3_c | If response to smear_benign_fu3 = 88, describe smear (cytological) findings | String |  |  |
| 809 | smear_fu3_d | Date of smear obtained at fu3 | Date | YYYY-MM-DD  (99 for any or all missing components – Y/M/D) |  |
| 810 | hpv_ fu3 | Woman received HPV testing at follow-up | Categorical | 0 = no  1 = yes  99 = not recorded | **If response hpv_ fu3 = 0, go to bx_fu3** |
| 811 | hpv_res_ fu3 | Result of HPV test at fu3 | Categorical | 0 = negative  1 = positive  99 = not recorded |  |
| 812 | hpv_fu3_c | If response to hpv_ fu3 = 1, please indicate the HPV sub-type identified | Numerical | Continuous variable  99 = not reported |  |
| 813 | bx_fu3 | Was a biopsy done at fu3 | Categorical | 0 = no  1 = yes  99 = not recorded | **If response to**  bx_fu3**= 0, go to** cryo_fu3 |
| 814 | bx_fu3_d | Date that the biopsy from fu3 was obtained | Date | YYYY-MM-DD  (99 for any or all missing components – Y/M/D) |  |
| 815 | bx_cin_fu3 | Biopsy result at fu3, presence of neoplasia | Categorical | 0 = No neoplasia  1 = CIN1  2 = CIN2  3 = CIN3  4 = ICC  5 = ASCUS  99 = not recorded |  |
| 816 | bx_benign_fu3 | Smear results at fu3, presence of benign disease  (select all relevant) | Categorical | 0 = No benign disease  1 = HPV  2 = cervicitis  88 = other  99 = not recorded |  |
| 817 | bx_fu3_c | If response to bx_benign_fu3 = 88, explain the histological findings | String |  |  |
| 818 | treat_fu3 | Did the woman receive treatment at fu3 | Categorical | 0 = no  1 = yes  99 = not recorded |  |
| 819 | cryo_fu3 | Received cryotherapy at fu3 | Categorical | 0 = no  1 = yes  99 = not recorded | **If response to cryo_fu3 = 0, go to leep_fu3** |
| 820 | cryo_fu3_d | Date that Cryo_fu3 was received | Date | YYYY-MM-DD  (99 for any or all missing components – Y/M/D) |  |
| 821 | leep_fu3 | LEEP done at fu3 | Categorical | 0 = no  1 = yes  99 = not recorded | **If response to leep_fu3 = 0, go ref_fu3** |
| 822 | leep_fu3_d | Date that leep_fu3 was performed | Date | YYYY-MM-DD  (99 for any or all missing components – Y/M/D) |  |
| 823 | leep_cin_fu3 | Histology results from leep_fu3, presence of neoplasia | Categorical | 0 = No neoplasia  1 = CIN1  2 = CIN2  3 = CIN3  4 = ICC  5 = ASCUS  99 = not recorded |  |
| 824 | leep_benign_fu3 | Histology results from leep_fu3, presence of benign disease  (select all relevant) | Categorical | 0 = No benign disease  1 = HPV  2 = cervicitis  88 = other  99 = not recorded |  |
| 825 | leep_fu3_c | If response to leep_benign_fu3 = 88, please describe (histological) findings | String |  |  |
| 826 | ref_fu3 | Referral made at fu3 | Categorical | 0 = no referral made  1 = referral for further screening  2 = referral for treatment precancer  3 = referral for treatment of suspected cancer  88 = other  99 = not recorded | **If response to ref_fu3 = 0, 1, 2, 88, 99, go to follow-up 2**  **If response to ref_fu3 = 3, go to Cascade step 8** |
| 827 | ref_fu3 | If response to ref_fu3 = 88, please explain | String |  |  |
| 828 | ref_date_fu3 | Date referral at follow-up 1 obtained | Date | YYYY-MM-DD  (99 for any or all missing components – Y/M/D) |  |

| **Follow-up4** | | | | | |
| --- | --- | --- | --- | --- | --- |
| 900 | fu | Did woman receive fourth follow-up visit (fu4) | Categorical | 0 = no  1 = yes  99 = not recorded | **If response to fu = 0, go to cascade step 7 “Cancer”** |
| 901 | screenby_fu4 | Person performing fourth follow-up | Categorical | 1 = nurse  2 = lay health worker  3 = doctor other than gynecologist  4 = gynecologist  99 = not recorded |  |
| 902 | via_fu4 | Did the woman receive VIA at her fourth follow-up visit | Categorical | 0 = no  1 = yes  99 = not recorded | **If response = 0, go to smear_fu4** |
| 903 | via_res_fu4 | VIA result at fu4 | Categorical | 0 = negative  1 = positive  2 = indeterminate  3 = suspicious of cancer  99 = not recorded | **In treated women, _fu4 will inform the cascade step “treatment outcomes”**  **In all other women it will inform the cascade step “re-examine”**  **If response via_res_fu4 = 0, go to follow-up 2** |
| 904 | via_fu4_d | Date via_res_fu4 obtained | Date | YYYY-MM-DD  (99 for any or all missing components – Y/M/D) |  |
| 905 | smear_fu4 | Did the woman receive a smear at fu4 | Categorical | 0 = no  1 = yes  99 = not recorded | **If response to Smear_fu4 = 0, go to HPV_fu4** |
| 906 | smear_cin_fu4 | Smear results at fu4, presence of neoplasia | Categorical | 0 = No neoplasia +/- benign disease  1 = CIN1  2 = CIN2  3 = CIN3  4 = ICC  5 = ASCUS  99 = not recorded |  |
| 907 | smear_benign_fu4 | Smear results at fu4, presence of benign disease  (select all relevant) | Categorical | 0 = No benign disease +/- CIN  1 = HPV  2 = cervicitis  88 = other  99 = not recorded |  |
| 908 | smear_fu4_c | If response to smear_benign_fu4 = 88, describe smear (cytological) findings | String |  |  |
| 909 | smear_fu4_d | Date of smear obtained at fu4 | Date | YYYY-MM-DD  (99 for any or all missing components – Y/M/D) |  |
| 910 | hpv_ fu4 | Woman received HPV testing at follow-up | Categorical | 0 = no  1 = yes  99 = not recorded | **If response hpv_ fu4 = 0, go to bx_fu4** |
| 911 | hpv_res_ fu4 | Result of HPV test at fu4 | Categorical | 0 = negative  1 = positive  99 = not recorded |  |
| 912 | hpv_fu4_c | If response to hpv_ fu4 = 1, please indicate the HPV sub-type identified | Numerical | Continuous variable  99 = not reported |  |
| 913 | bx_fu4 | Was a biopsy done at fu4 | Categorical | 0 = no  1 = yes  99 = not recorded | **If response to**  bx_fu4**= 0, go to** cryo_fu4 |
| 914 | bx_fu4_d | Date that the biopsy from fu4 was obtained | Date | YYYY-MM-DD  (99 for any or all missing components – Y/M/D) |  |
| 915 | bx_cin_fu4 | Biopsy result at fu4, presence of neoplasia | Categorical | 0 = No neoplasia  1 = CIN1  2 = CIN2  3 = CIN3  4 = ICC  5 = ASCUS  99 = not recorded |  |
| 916 | bx_benign_fu4 | Smear results at fu4, presence of benign disease  (select all relevant) | Categorical | 0 = No benign disease  1 = HPV  2 = cervicitis  88 = other  99 = not recorded |  |
| 917 | bx_fu4_c | If response to bx_benign_fu4 = 88, explain the histological findings | String |  |  |
| 918 | treat_fu4 | Did the woman receive treatment at fu4 | Categorical | 0 = no  1 = yes  99 = not recorded |  |
| 919 | cryo_fu4 | Received cryotherapy at fu4 | Categorical | 0 = no  1 = yes  99 = not recorded | **If response to cryo_fu4 = 0, go to leep_fu4** |
| 920 | cryo_fu4_d | Date that Cryo_fu4 was received | Date | YYYY-MM-DD  (99 for any or all missing components – Y/M/D) |  |
| 921 | leep_fu4 | LEEP done at fu4 | Categorical | 0 = no  1 = yes  99 = not recorded | **If response to leep_fu4 = 0, go ref_fu4** |
| 922 | leep_fu4_d | Date that leep_fu4 was performed | Date | YYYY-MM-DD  (99 for any or all missing components – Y/M/D) |  |
| 923 | leep_cin_fu4 | Histology results from leep_fu4, presence of neoplasia | Categorical | 0 = No neoplasia  1 = CIN1  2 = CIN2  3 = CIN3  4 = ICC  5 = ASCUS  99 = not recorded |  |
| 924 | leep_benign_fu4 | Histology results from leep_fu4, presence of benign disease  (select all relevant) | Categorical | 0 = No benign disease  1 = HPV  2 = cervicitis  88 = other  99 = not recorded |  |
| 925 | leep_fu4_c | If response to leep_benign_fu4 = 88, please describe (histological) findings | String |  |  |
| 926 | ref_fu4 | Referral made at fu4 | Categorical | 0 = no referral made  1 = referral for further screening  2 = referral for treatment precancer  3 = referral for treatment of suspected cancer  88 = other  99 = not recorded | **If response to ref_fu4 = 0, 1, 2, 88, 99, go to follow-up 2**  **If response to ref_fu4 = 3, go to Cascade step 8** |
| 927 | ref_fu4 | If response to ref_fu4 = 88, please explain | String |  |  |
| 928 | ref_date_fu4 | Date referral at follow-up 1 obtained | Date | YYYY-MM-DD  (99 for any or all missing components – Y/M/D) |  |

| **Cascade Step 7: Cancer** | | | | | |
| --- | --- | --- | --- | --- | --- |
| 1000 | cancer | Woman has developed cervical cancer during study period | Categorical | 1 = yes  2 = no  99 = not recorded |  |
| 1001 | cx_treat_eua | Did the woman receive and examination under anesthesia (EUA) | Categorical | 1 = yes  2 = no  99 = not recorded |  |
| 1002 | cx_treat_eua_d | Date of EUA | Date | YYYY-MM-DD  (99 for any or all missing components – Y/M/D) |  |
| 1003 | cx_treat_cone | Did the woman receive a cone biopsy | Categorical | 1 = yes  2 = no  99 = not recorded |  |
| 1004 | cx_treat_cone_d | Date of cone biopsy | Date | YYYY-MM-DD  (99 for any or all missing components – Y/M/D) |  |
| 1005 | cx_treat_hyst | Did the woman receive a simple hysterectomy | Categorical | 1 = yes  2 = no  99 = not recorded |  |
| 1006 | cx_treat_hyst_d | Date of simple Hysterectomy | Date | YYYY-MM-DD  (99 for any or all missing components – Y/M/D) |  |
| 1007 | cx_treat_radhyst | Did the woman receive a radical hysterectomy + lymph node dissection (LNs) | Categorical | 1 = yes  2 = no  99 = not recorded |  |
| 1008 | cx_treat_radhyst_d | Date of radical hysterectomy + lymph node dissection (LNs) | Date | YYYY-MM-DD  (99 for any or all missing components – Y/M/D) |  |
| 1009 | cx_treat_rtx | Did the woman receive radiotherapy | Categorical | 1 = yes  2 = no  99 = not recorded |  |
| 1010 | cx_treat_rtx_d | Date of radiotherapy | Date | YYYY-MM-DD  (99 for any or all missing components – Y/M/D) |  |
| 1011 | cx_treat _ctx | Did the woman receive chemotherapy | Categorical | 1 = yes  2 = no  99 = not recorded |  |
| 1012 | cx_treat _ctx _d | Date of chemotherapy | Date | YYYY-MM-DD  (99 for any or all missing components – Y/M/D) |  |
| 1013 | cx_treat _hosppal | Did the woman receive hospital palliation | Categorical | 1 = yes  2 = no  99 = not recorded |  |
| 1014 | cx_treat _hosppal_d | Date of hospital palliative therapy | Date | YYYY-MM-DD  (99 for any or all missing components – Y/M/D) |  |
| 1015 | cx_treat _homepal | Did the woman receive home palliation | Categorical | 1 = yes  2 = no  99 = not recorded |  |
| 1016 | cx_treat _homepal_d | Date of home palliative therapy | Date | YYYY-MM-DD  (99 for any or all missing components – Y/M/D) |  |
