# Supplementary material for: Cervical Cancer Screening Cascade for women living with HIV: A cohort study from Zimbabwe
Source: PLOS Glob Public Health. 2022 Feb 2;2(2):e0000156. doi: 10.1371/journal.pgph.0000156 (PMC9974171; doi:10.1371/journal.pgph.0000156)
Supplement: S3 Table — (DOCX) [file pgph.0000156.s007.docx]

**Supporting information 4: Results of the cervical cancer prevention cascade using the longitudinal approach**

| **Secondary cervical cancer prevention cascade** | **Number of women** | **Time to stage**  days, median (IQR) | **Cumulative incidence at 2 years***  % (95%CI) |
| --- | --- | --- | --- |
| **Screening arm** |  |  |  |
| Women in care | 1624 | 63 (28-146) | 85.4 (83.5-87.1) |
| Screened | 1385 | 63 (28-154) | 60.6 (58.1-62.9) |
| Screen-negative at first screening | 989 | 374 (361-424) | 44.9 (42.3-47.3) |
| Rescreened | 791 | 374 (363-427) | 40.6 (38.1-43.0) |
| Screen-negative at rescreening | 713 |  |  |
|  |  |  |  |
| **Preventative treatment arm** |  |  |  |
| Screen-positive at first screening | 396 | 64.5 (28-133) |  |
| Treated | 316 | 13 (0-37) | 79.5 (75.1-83.2) |
| Rescreened | 277 | 188 (181-223) | 61.7 (56.6-66.3) |
| Screen-negative at rescreening | 169 | 188 (182-231) | 36.1 (31.2-40.7) |
|  |  |  |  |

Longitudinal approach uses all individual patient level data available over the study period; ^*^Start stage for cumulative incidence in the screening arm was the date of women’s first ART clinic appointment, end stages are the indicated cascade stages occurring subsequently; ^§^start stage for cumulative incidence in the preventative treatment arm is the screen-positive date at first screening, end stages are the indicated cascade stages occurring subsequently; n: number of women, IQR: inter-quartile range, CI: confidence interval
