# Supplementary material for: Cervical Cancer Screening Cascade for women living with HIV: A cohort study from Zimbabwe
Source: PLOS Glob Public Health. 2022 Feb 2;2(2):e0000156. doi: 10.1371/journal.pgph.0000156 (PMC9974171; doi:10.1371/journal.pgph.0000156)
Supplement: S4 Table — (DOCX) [file pgph.0000156.s008.docx]

**Supporting information 7. Crude and adjusted hazard ratios for factors associated with screen-negativity after treatment**

|  | **Univariable analysis**  **n = 1251** | | | **Multivariable analysis**  **n = 1251** | | |
| --- | --- | --- | --- | --- | --- | --- |
|  | HR | 95% CI | P value | HR | 95% CI | P value |
| **Age, years** |  |  |  |  |  |  |
| <25 | 1.13 | 0.65-1.96 |  | 1.26 | 0.65-2.48 |  |
| 25-44 | 1 | - | 0.216 | 1 | - | 0.075 |
| >45 | 1.78 | 0.87-3.62 |  | 2.33 | 1.03-5.26 |  |
| **Relationship** |  |  |  |  |  |  |
| Single | 0.81 | 0.56-1.17 |  |  |  |  |
| Relationship | 1 | - | 0.413 |  |  |  |
| Separated | 0.81 | 0.55-1.18 |  |  |  |  |
| **Employment** |  |  |  |  |  |  |
| Unemployed | 1 | - |  | 1 | - |  |
| Employed | 1.00 | 0.59-1.14 | 0.001 | 0.84 | 0.59-1.19 | 0.122 |
| Student | 0.09 | 0.01-0.64 |  | 0.15 | 0.02-1.08 |  |
| **Parity** |  |  |  |  |  |  |
| 0 |  |  |  |  |  |  |
| 1 to 3 | 0.94 | 0.56-1.59 | 0.841 | 0.99 | 0.51-1.91 | 0.991 |
| >3 | 1.06 | 0.58-1.95 |  | 0.96 | 0.45-2.04 |  |
| **HIV RNA viral load at VIA screening. copies/ mm^3^** |  |  |  |  |  |  |
| <50 | 1 | - |  | 1 | - |  |
| 50-1000 | 1.16 | 0.70-1.92 | 0.275 | 1.08 | 0.64-1.82 | 0.546 |
| >1000 | 1.31 | 0.94-1.82 |  | 1.22 | 0.86-1.75 |  |
| **Year enrolled, year** |  |  |  |  |  |  |
| Early years (2012-2014) | 1 | - | 0.533 | 1 | - | 0.316 |
| Later years (2015-2017) | 0.90 | 0.66-1.24 |  | 0.84 | 0.59-1.19 |  |

Copies/ mm3: copies in a cubic millimetre; CI: confidence interval; HIV: human immunodeficiency virus; HR: hazards ratio; n: number of women; n: number of women; RNA: Ribonucleic acid; VIA: visual inspection with acetic acid; n: number of women.
